# Chemical Induction of MYC Protein Degradation via MYC–MAX Disruption and 20S Proteasome Activation

**DOI:** 10.1021/acschembio.6c00258

**Published:** 2026-06-19

**Authors:** Miracle O. Olatunde, Jetze J. Tepe

**Affiliations:** † Department of Chemistry, 2358 University of Virginia, Charlottesville, Virginia 22904, United States; ‡ University of Virginia, Comprehensive Cancer Center, Charlottesville, Virginia 22904, United States

## Abstract

The MYC oncoprotein is a master regulator of cell growth and transcriptional amplification and is aberrantly overexpressed in a broad spectrum of human cancers, including colorectal carcinoma. Despite its central role in tumorigenesis, MYC has remained pharmacologically intractable due to its intrinsically disordered architecture, which lacks persistent small-molecule binding pockets. Here, we report a chemical biology strategy that exploits MYC’s structural disorder as a therapeutic vulnerability. By combining small-molecule disruption of the MYC–MAX protein–protein interaction with pharmacological activation of the 20S proteasome, we induce rapid and pronounced depletion of MYC in MYC-dependent colorectal cancer cell lines. MYC loss is proteasome-dependent and persists following knockdown of FBXW7, indicating a degradation mechanism distinct from canonical SCF–FBXW7-mediated turnover and consistent with direct 20S proteasomal degradation. Dual treatment also suppresses MYC-driven transcriptional programs and significantly enhances apoptotic cell death. Collectively, these findings establish a framework in which protein–protein interaction inhibition sensitizes intrinsically disordered oncoproteins to 20S proteasome-mediated degradation. This work expands the therapeutic landscape for MYC-driven malignancies and highlights proteasome activation as a complementary strategy for targeting structurally disordered cancer drivers.

## Graphical abstract

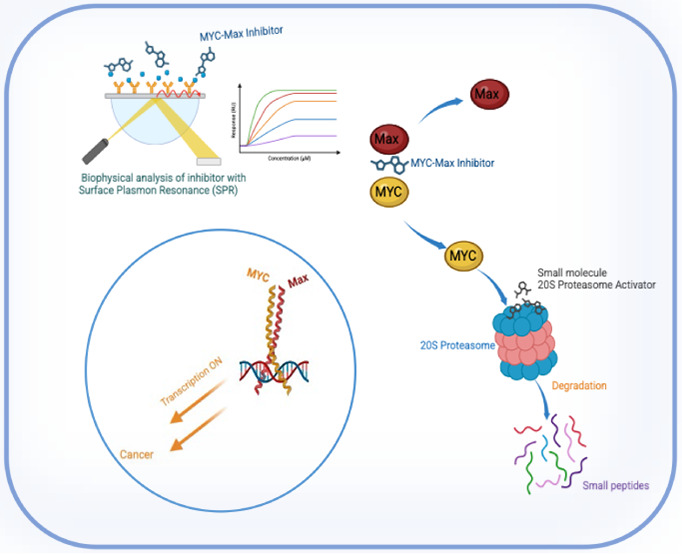

## Introduction

MYC (cMyc) is a key oncogenic driver, with amplification of the MYC gene occurring in approximately two-thirds of human cancers.^1^ MYC regulates the transcription of a broad network of genes that control angiogenesis, cellular proliferation, and survival, thereby playing a pivotal role in tumorigenesis and establishing MYC as a highly attractive target for therapeutic intervention.^2–4^ Under normal physiological conditions, MYC is predominantly degraded through the ubiquitin–proteasome system (UPS) governed by the 26S proteasome.^5,6^ MYC’s ubiquitination is tightly regulated by sequential phosphorylation events, in which phosphorylation of serine 62 by cyclin-dependent kinases (CDK) primes MYC for subsequent phosphorylation at threonine 58 by GSK-3β.^7,8^ These modifications create a phospho-degron that is recognized by the E3 ubiquitin ligase FBXW7,^9^ leading to MYC ubiquitination and subsequent 26S proteasome-mediated degradation. In cancer cells, disruption of this tightly regulated phosphorylation–ubiquitination cascade, through aberrant kinase signaling, actions of deubiquitinases, or loss of FBXW7 function impairs MYC turnover, and leads to pathological stabilization and accumulation.^7,8,10,11^

Several challenges attenuate the effective targeting of MYC in cancer. MYC protein has a highly disordered structure, historically rendering it “undruggable” as the absence of stable secondary or tertiary structure precludes the formation of well-defined binding pockets amenable to conventional small-molecule inhibition.^12,13^ This structural plasticity underscores the need for innovative therapeutic strategies that target MYC directly or indirectly by modulating its interactions or stability. Additionally, MYC exerts its oncogenic function through a high affinity protein–protein interaction (PPI) with its obligate partner protein MAX, an interaction that induces a transition from disorder to a more ordered, stable α-helical conformation within the basic helix–loop–helix leucine zipper (bHLH-LZ) domain.^14–17^ Together, these features not only complicate pharmacological targeting but also promote pathological MYC stabilization, thereby exacerbating its accumulation and amplifying downstream transcriptional programs that drive cancer progression.

One strategy to therapeutically target MYC involves the use of small molecules that disrupt MYC obligate dimerization with MAX suppressing MYC-driven transcription.^18–20^ Although these inhibitors transiently reduce MYC-dependent transcription in vitro, inhibition of the MYC–MAX interaction alone produces only short-lived effects in vivo.^21,22^ This limited durability reflects both rapid compound clearance and the high-affinity, dynamic nature of the MYC–MAX interaction, which allows MYC to quickly reassociate with MAX once inhibitor levels decline. This highlights the need for strategies that move beyond reversible dimerization blockade and instead promote sustained suppression or irreversible clearance of MYC.

Recently, Young et al. reported a proteolysis-targeting chimera (PROTAC) that induces MYC degradation by recruiting an E3 ubiquitin ligase to the 26S proteasome while simultaneously disrupting MYC–MAX dimerization.^23^ This study demonstrates that interference with MYC–MAX complex formation can be combined with targeted manipulation of cellular degradation pathways. The authors further observed partial truncation of MYC at the *N*-terminal transactivation domain, leaving the *C*-terminal region intact and potentially capable of retaining transcriptional activity. These observations motivated the exploration of complementary but distinct proteasome-centered strategy, focused on leveraging 20S proteasome activity to directly engage the vital intrinsically disordered regions of MYC.

The 26S proteasome degradation pathway is a highly intricate and tightly regulated system composed of multiple coordinated processes and components that collectively govern protein turnover.^24^ Within this broader proteostasis network, the 20S proteasome plays a pivotal and mechanistically distinct role, mediating the selective degradation of intrinsically disordered proteins (IDPs) in a ubiquitin- and ATP-independent manner. Serving as the catalytic core of the 26S proteasome complex, the 20S proteasome comprises four stacked heptameric rings made up of two outer α-rings and two inner β-ring, where the proteolytic activity is mediated by the *N*-terminal threonine residues of the β-subunits.^25–27^ In contrast to the 26S proteasome, the 20S proteasome core particle mediates protein degradation independently of ubiquitination, instead recognizing substrates based on intrinsic structural features.^24,28^ The 20S proteasome core particle contains a highly restricted substrate entry portal, defined by an 13 Å axial gate on the outer α-rings formed by the *N*-terminal tails.^29^ Access to the proteolytic core is limited to proteins that can thread through this narrow opening,^30^ making the highly intrinsically disordered monomeric MYC protein an ideal 20S proteasome substrate.^31^ The 20S proteasome degradation pathway can be pharmacologically enhanced by small-molecule activation of the 20S proteasome, thereby accelerating proteolysis of intrinsically disordered proteins.^32–37^ This possibility suggests a strategy to selectively reduce MYC protein levels in cancer cells. Considering the ordered structure of the intact MYC–MAX complex, this heterodimer itself is unlikely to be a 20S proteasome substrate. We therefore hypothesize that disruption of the MYC–MAX interaction will liberate intrinsically disordered, monomeric MYC, rendering it susceptible to direct 20S proteasome-mediated degradation.

Herein, we introduce a complementary but distinct therapeutic strategy to target the degradation of MYC that combines two orthogonal approaches: disruption of proteolytically stable and structurally ordered MYC–MAX complex and clearance of monomeric MYC through 20S proteasome enhancement. By leveraging a small molecule MYC–MAX inhibitor in conjunction with 20S proteasome activators, we aim to simultaneously accelerate MYC’s clearance and suppress its transcriptional activity and, thereby blocking oncogenic signaling and cancer cell proliferation.

## Results

### Screening of MYC–MAX Inhibitors by Surface Plasmon Resonance Analysis

MYC is stabilized through heterodimerization with MAX through its highly disordered basic helix–loop–helix leucine zipper (bHLHLZ) domain (Figure 1A). This heterodimer binds DNA at promoter regions, as revealed by previously reported crystal structures (Figure 1B). Several small-molecule inhibitors have previously been identified that disrupt MYC–MAX heterodimerization.^18–20,38^ To validate direct binding to MYC and assess their ability to interfere with MYC–MAX complex formation, we evaluated a panel of well-established MYC–MAX inhibitors, including the prototypical compounds 10058-F4,^20^ 10074-G5,^19^ and MYCMI-11 ^39^ (Figure 1C).

**Figure 1:**
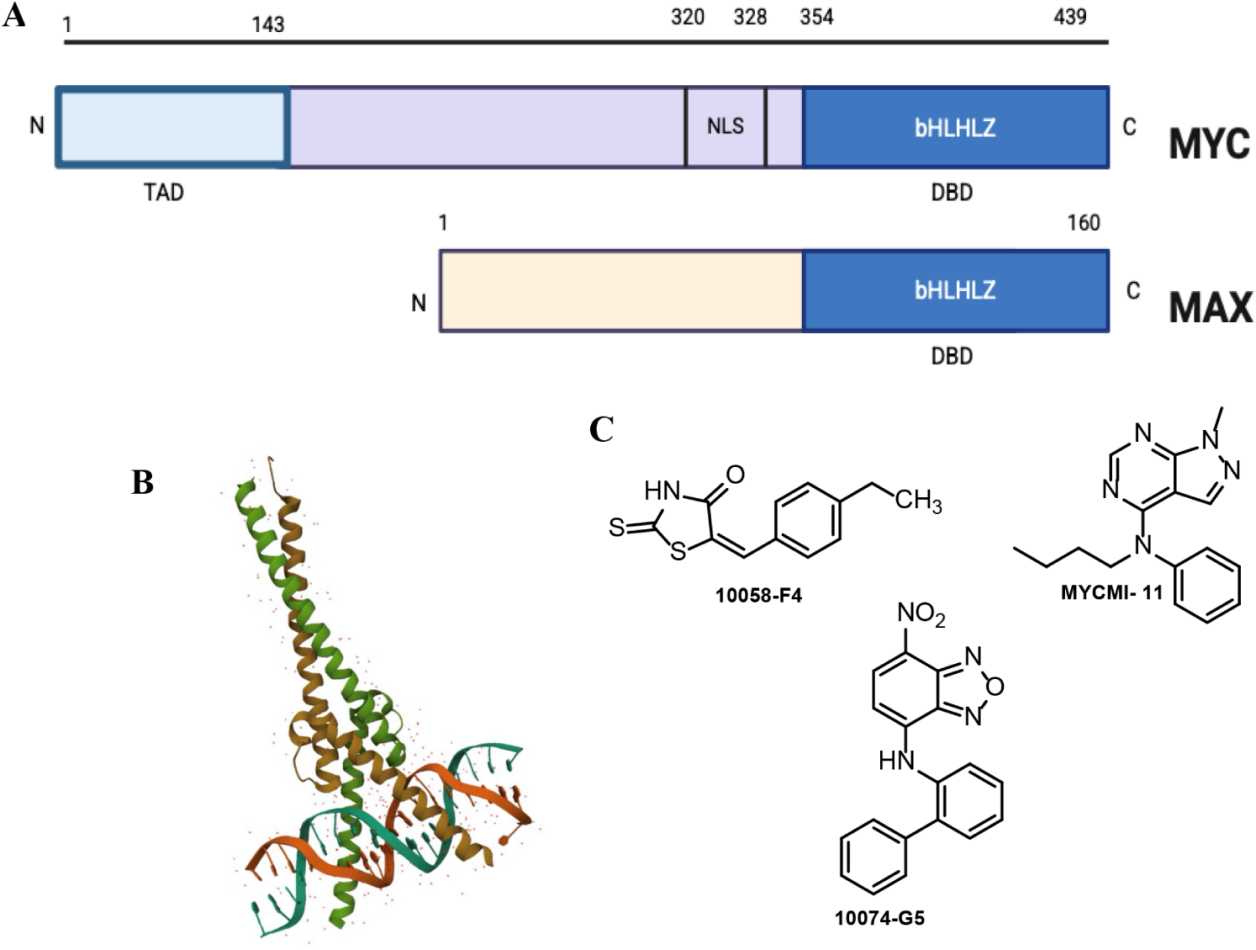
Structural overview of MYC–MAX dimerization and representative small-molecule inhibitors. (A) Cartoon illustration of MYC and MAX highlighting their basic helix–loop–helix leucine zipper (bHLHLZ) domains, DNA binding domain (DBD) and transactivational domain (TAD). (B) Crystal structure of MYC and MAX bound to DNA (PDB: 1NKP).^12^ (C) Chemical structures of small-molecule inhibitors reported to disrupt MYC–MAX heterodimerization, including 10058-F4, MYCMI-11, and 10074-G5.

Direct MYC binding was characterized using surface plasmon resonance (SPR) assay to obtain real-time kinetic and affinity data Figure S1). Recombinant GST-tagged MYC (amino acids 330–439 of human c-MYC, corresponding to the bHLH-LZ domain responsible for MAX binding) was immobilized on CM5 sensor chips through anti-GST capture. Increasing concentrations of each small molecule inhibitor were injected over the surface to monitor binding interactions. The observed equilibrium dissociation constants (K_D_) for the tested inhibitors are summarized in Table 1.

**Table 1:** K_D_ Values of MYC–MAX Inhibitors Binding to the bHLH-LZ of MYC^a^

Compound	K_D_ (μM)
10058-F4	37 ± 7.6
MYCMI-11	24 ± 1.1
10074-G5^b^	–

a Data represent mean ± SEM from three independent experiments.

b Steady state binding was not achieved at higher analyte concentrations due to solubility limitations.

Given its relatively superior binding affinity for MYC (K_D_ = 24 μM) as measured by SPR (Figure S1), along with its favorable solubility and synthetic accessibility, we selected MYCMI-11 (previously identified in a high throughput screen) for further biological evaluation and combination studies with 20S proteasome activators.^39^

### MYCMI-11 Does Not Enhance 20S Proteasome Activity

MYCMI-11 was synthesized according to literature procedures for the preparation of the 4-chloro-1-methyl-1H-pyrazolo­[3,4-*d*]­pyrimidine core, followed by nucleophilic aromatic substitution at the 4-chloro position with butylaniline.^40^ We then evaluated whether MYCMI-11 directly modulates 20S proteasome activity in vitro. Proteasome activation was assessed using fluorogenic 7-amino-4-methylcoumarin (AMC) tagged peptide degradation assay with recombinant human 20S proteasome. Substrate turnover was monitored using 20 μM each of Suc-LLVY-AMC, Boc-LLR-AMC, and Z-LLE-AMC, which report on the chymotrypsin-like, trypsin-like, and caspase-like proteolytic activities, respectively from fluorescence unit over time. Initial rates were determined from the linear portion of the progress curve. MYCMI-11 was tested across a concentration range (80–0.3125 μM) encompassing and exceeding its reported cellular activity and compared to the well-established 20S proteasome activators TCH-165^36^ and SS-4-15^37^ (Figure 2A).

**Figure 2:**
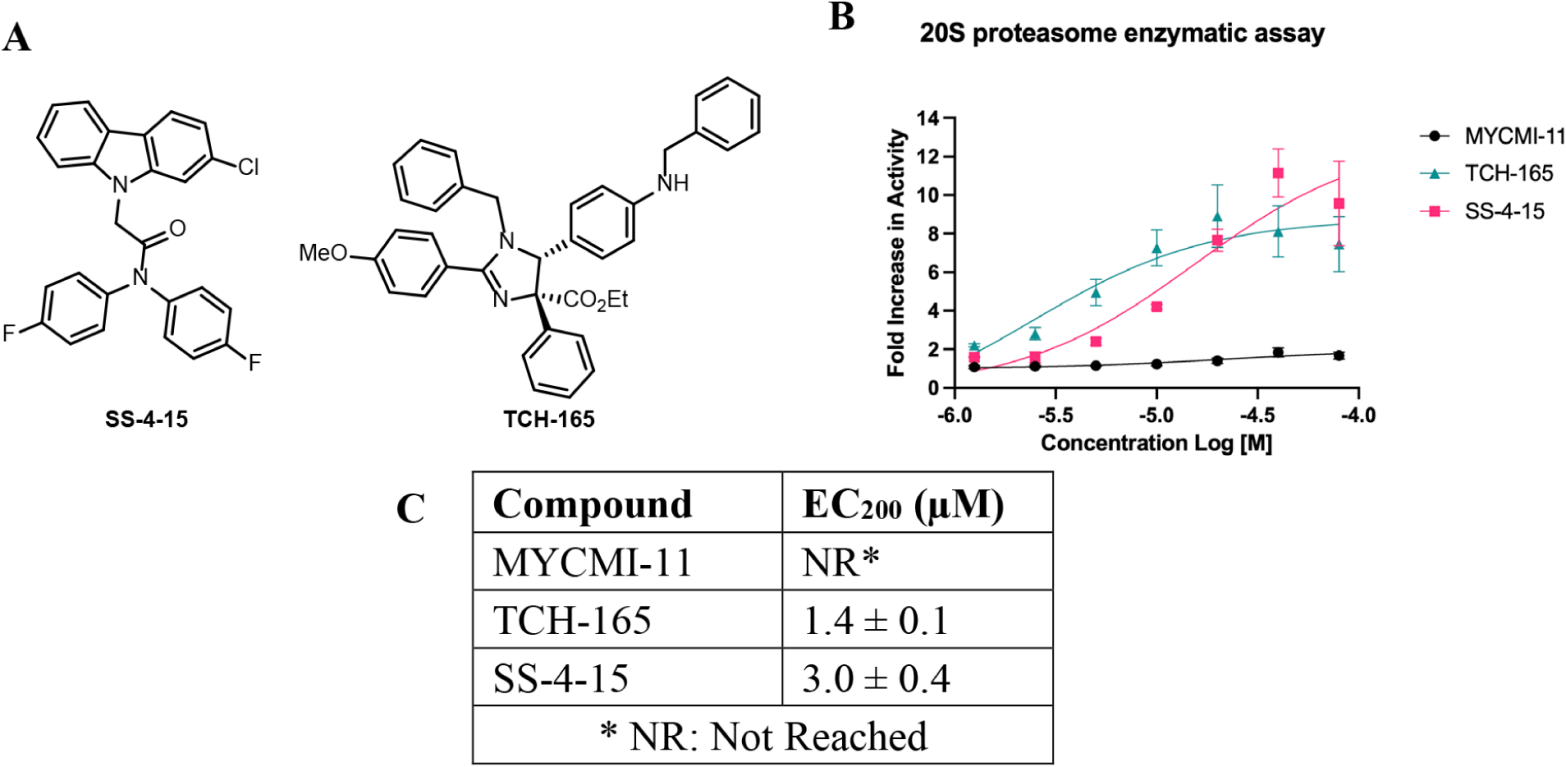
Targeting MYC–MAX and 20S proteasome-mediated peptide degradation. (A) Structure of small molecule 20S proteasome activators: TCH-165 and SS-4-15. (B) Dose-dependent enhancement of 20S proteasome-mediated small peptide degradation of fluorogenic substrates: Suc-LLVY-AMC for chymotrypsin-like (CT-L), Boc-LRR-AMC for trypsin-like (Tryp-L), and Z-LLE-AMC for caspase-like (Casp-L) activities of 20S proteasome catalytic sites following treatment with MYC–MAX inhibitor MYCMI-11, and 20S proteasome activators: TCH-165 and SS-4-15. Data represent mean ± SEM from three independent experiments. (C) Experimental EC_200_ values calculated from (B) for the indicated compounds from in vitro 20S proteasome activation assay.

The concentration of the drug required for induction of 2-fold increase in 20S proteasomal activity (EC_200_) was determined by fitting the relative fluorescence units and concentrations into a four-parameter dose–response curve (Figure 2B). In contrast to these positive controls (TCH-165 and SS-4-15), which produced robust enhancement of the rate of 20S proteasome proteolysis of the substrates consistent with their reported results (Figure 2C), MYCMI-11 did not enhance substrate turnover above baseline for any proteolytic activity. These results demonstrate that MYCMI-11 does not enhance the proteolytic activity of the 20S proteasome, indicating that its primary biological activity is not mediated through direct 20S proteasome enhancement.

### Monomeric MYC Is Directly Degraded by the 20S Proteasome

To determine whether MYC is a direct substrate of the 20S proteasome, we evaluated the ability of purified human 20S proteasome to degrade monomeric MYC in vitro. 20S proteasome (10 nM) was preincubated with 10 μM of known 20S proteasome enhancers including, SS-4-15 and TCH-165 for 45 min, followed by the addition of purified MYC protein (79 nM) and incubation at 37 °C for 4 h. Proteasome inhibitor bortezomib (10 μM) was used as a control. Both SS-4-15 and TCH-165 significantly decreased the amount of MYC protein remaining compared to proteasome alone (Figure 3), whereas bortezomib blocked MYC degradation, confirming proteasome-dependent proteolysis. In contrast, the MYC–MAX inhibitor MYCMI-11 did not promote MYC degradation in this assay, consistent with our observations in the fluorogenic peptide degradation assay (Figure 2B). Together, these findings demonstrate that monomeric MYC is directly degraded by the 20S proteasome and that this process can be potentiated by small-molecule 20S activators, supporting pharmacological proteasome enhancement as a strategy to reduce MYC protein levels.

**Figure 3:**
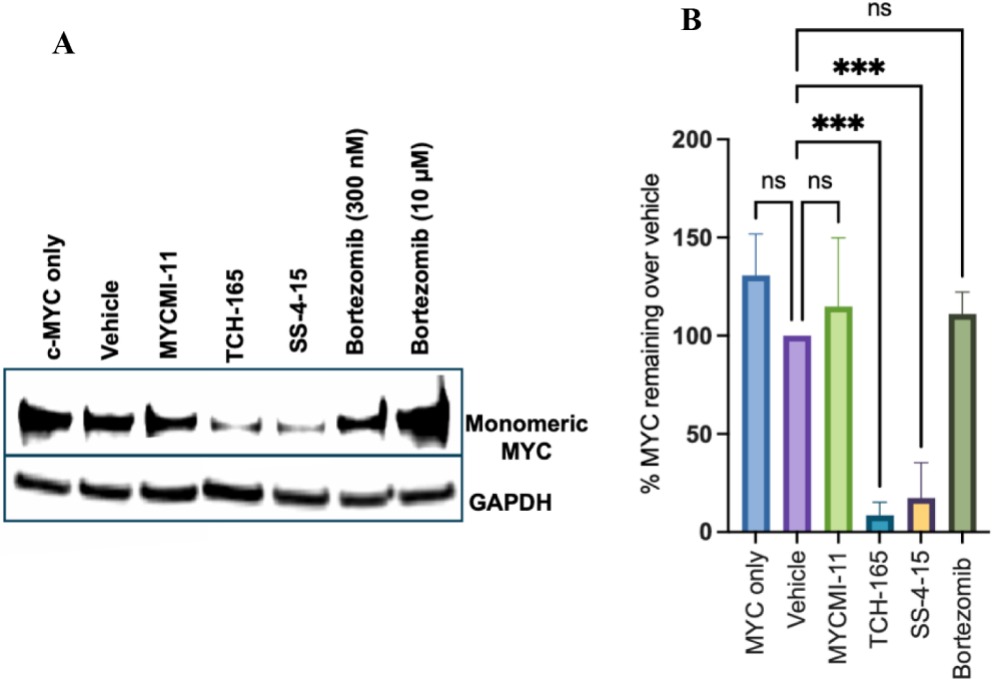
In vitro monomeric MYC degradation through 20S proteasome activation. (A) Purified human 20S proteasome (10 nM) was incubated with purified monomeric MYC (79 nM) at 37 °C for 4 h in the presence of 10 μM of proteasome activators including SS-4-15, TCH-165, or MYC–MAX inhibitor, MYCMI-11. Bortezomib (300 nM and 10 μM) was included as a proteasome inhibitor control. MYC levels were analyzed by immunoblotting, with purified GAPDH used as a loading control, and densitometric quantification is shown in accompanying graph (B). Error bars represent mean ± SEM from three independent experiments (*n* = 3). Statistical significance was determined by one-way ANOVA with Dunnett’s multiple-comparisons test relative to vehicle-treated control (*p < 0.05, **p < 0.01, ***p < 0.001).

### Dual Treatment of Colon Cancer Cells with MYC–MAX Inhibitor and 20S Proteasome Enhancers Reduces MYC Protein Levels in a Proteasome-Dependent Manner

Colon cancer is one of the malignancies in which MYC is frequently overexpressed.^1,41^ Because MYC exists in cells as a heterodimer with MAX, we hypothesized that disruption of the MYC–MAX interaction would expose intrinsically disordered regions of monomeric MYC, thereby increasing its susceptibility to ubiquitin-independent 20S proteasomal degradation (as shown in Figure 3). Based on this hypothesis, we investigated whether simultaneous disruption of MYC–MAX dimerization and activation of the 20S proteasome could cooperatively enhance MYC turnover. To test this, the MYC–MAX inhibitor MYCMI-11 was evaluated alone and in combination with the 20S proteasome activators TCH-165 and SS-4-15 in the high-MYC expressing colorectal cancer cell lines HCT-116 and HT-29.

Treatment with MYCMI-11 or either proteasome activator alone produced minimal changes in steady-state MYC protein levels over a 4-h period. However, in HCT-116 cells, cotreatment with MYCMI-11 (5 μM) and TCH-165 (2 μM) resulted in a significant reduction in MYC levels (Figure 4A). Bortezomib (300 nM), a proteasome inhibitor, was used to validate proteasome-dependent MYC degradation. Increasing the concentration of the proteasome activators to 5 μM in HT-29 cells, in combination with MYCMI-11 (5 μM), resulted in more than a 50% reduction in MYC protein levels with TCH-165 and an approximately 30% decrease with SS-4-15 relative to either agent alone or vehicle (Figure 4B). These findings are consistent with an additive effect between MYC–MAX inhibition and 20S proteasome activation. Next, we investigated whether the observed reduction in MYC levels was proteasome-dependent. Pretreatment of HT-29 cells with the proteasome inhibitor bortezomib for 1 h prior to combined treatment with the MYC–MAX inhibitor and 20S proteasome activator markedly attenuated MYC degradation (Figure 4C), supporting a proteasome-dependent mechanism underlying the enhanced MYC turnover observed with dual treatment. Collectively, the data support a model in which disruption of MYC–MAX dimerization increases the susceptibility of MYC to 20S proteasome-mediated degradation, providing a complementary chemical strategy to reduce MYC stability in cancer cells.

**Figure 4:**
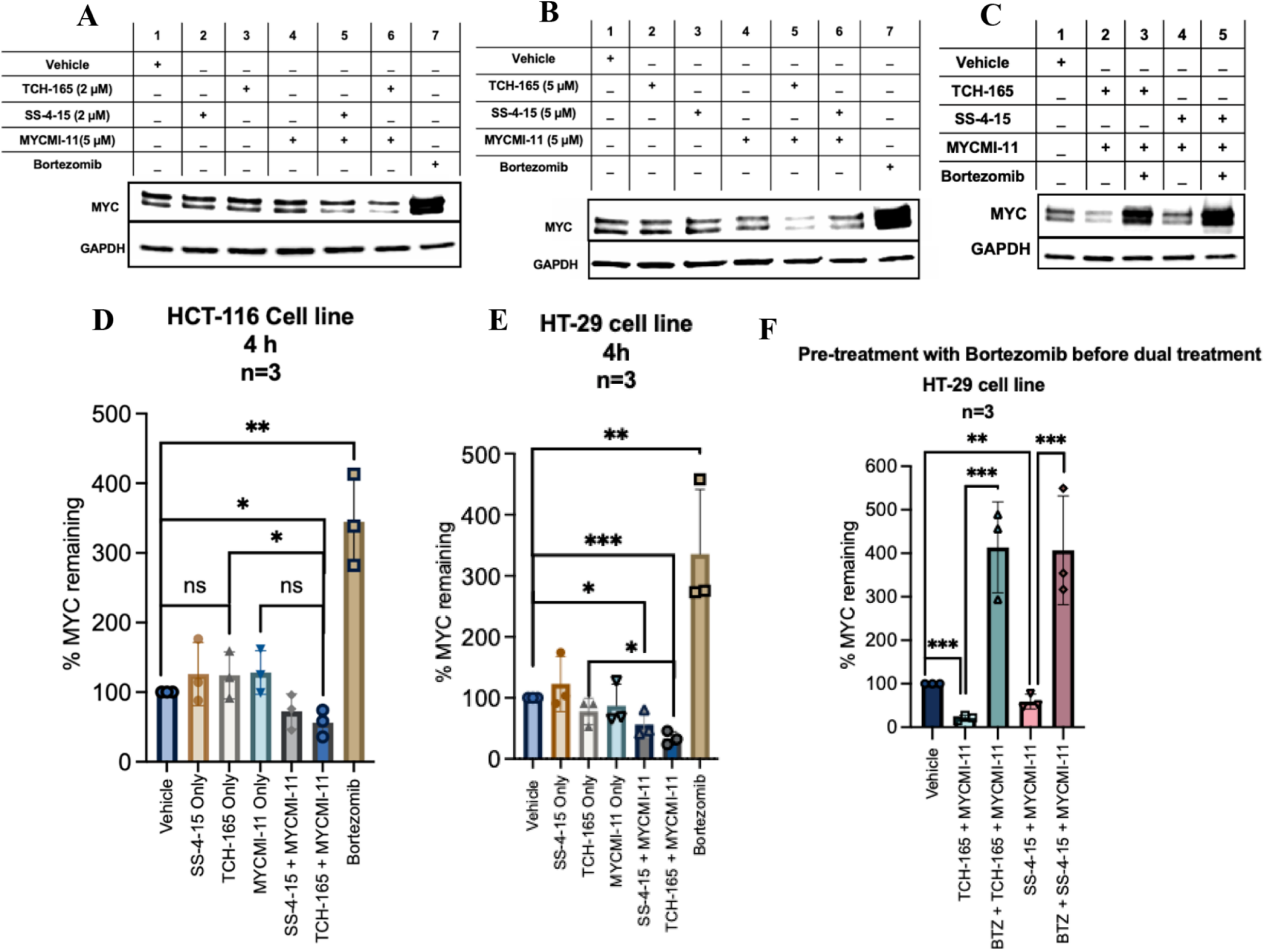
Combined MYC–MAX inhibition and 20S proteasome activation reduces MYC protein levels in colorectal cancer cells in a proteasome-dependent manner. (A) HCT-116 cells were treated for 4 h with MYCMI-11 (5 μM), TCH-165 (2 μM), SS-4-15 (2 μM), or the indicated combinations. (B) HT-29 cells were treated for 4 h with MYCMI-11 (5 μM), TCH-165 (5 μM), SS-4-15 (5 μM), or the indicated combinations. Bortezomib, BTZ, (300 nM) was included as a proteasome inhibitor control to validate proteasome-dependent MYC degradation. (C) HT-29 cells were pretreated with bortezomib (300 nM) for 1 h prior to combination treatment with MYCMI-11 (5 μM) plus either TCH-165 (5 μM) or SS-4-15 (5 μM). Total cell lysates were analyzed by immunoblotting for MYC (∼53 kDa), with GAPDH (∼36 kDa) as a loading control. (D–F) Densitometric quantification of MYC protein levels normalized to GAPDH and expressed relative to vehicle control is shown for HCT-116 (D), HT-29 (E), and bortezomib-pretreated HT-29 cells (F). Data are presented as mean ± SEM from three independent experiments (n = 3). Statistical significance was determined by one-way ANOVA with Dunnetts multiple-comparisons test versus vehicle control (*p < 0.05, **p < 0.01, ***p < 0.001).

### Dual Targeting of MYC with MYC–MAX Inhibitor and 20S Proteasome Activators Impairs MYC-Dependent Transcription

Given the central role of MYC as a transcription factor, we next assessed whether enhanced MYC degradation induced by combined MYC–MAX dimerization inhibition and 20S proteasome activation translates into suppression of MYC-dependent transcriptional activity. TCH-165 was chosen for further functional studies due to its enhanced reduction of cellular MYC protein levels (Figure 4). HCT-116 colon cancer cells stably expressing a luminescence-based MYC reporter were treated with increasing concentrations of MYCMI-11 (0.3–40 μM), TCH-165 (2 μM), or the combination of both agents for 16 h. Treatment with MYCMI-11 or TCH-165 alone had little effect on reporter activity. In contrast, the combination treatment produced significant, dose-dependent suppression of reporter signal compared to either agent alone. ICG-001 (0.15–20 μM), a small molecule inhibitor of β-catenin–CBP-dependent transcription,^42^ was included as a control for suppression of MYC-driven transcription (Figure 5).

**Figure 5:**
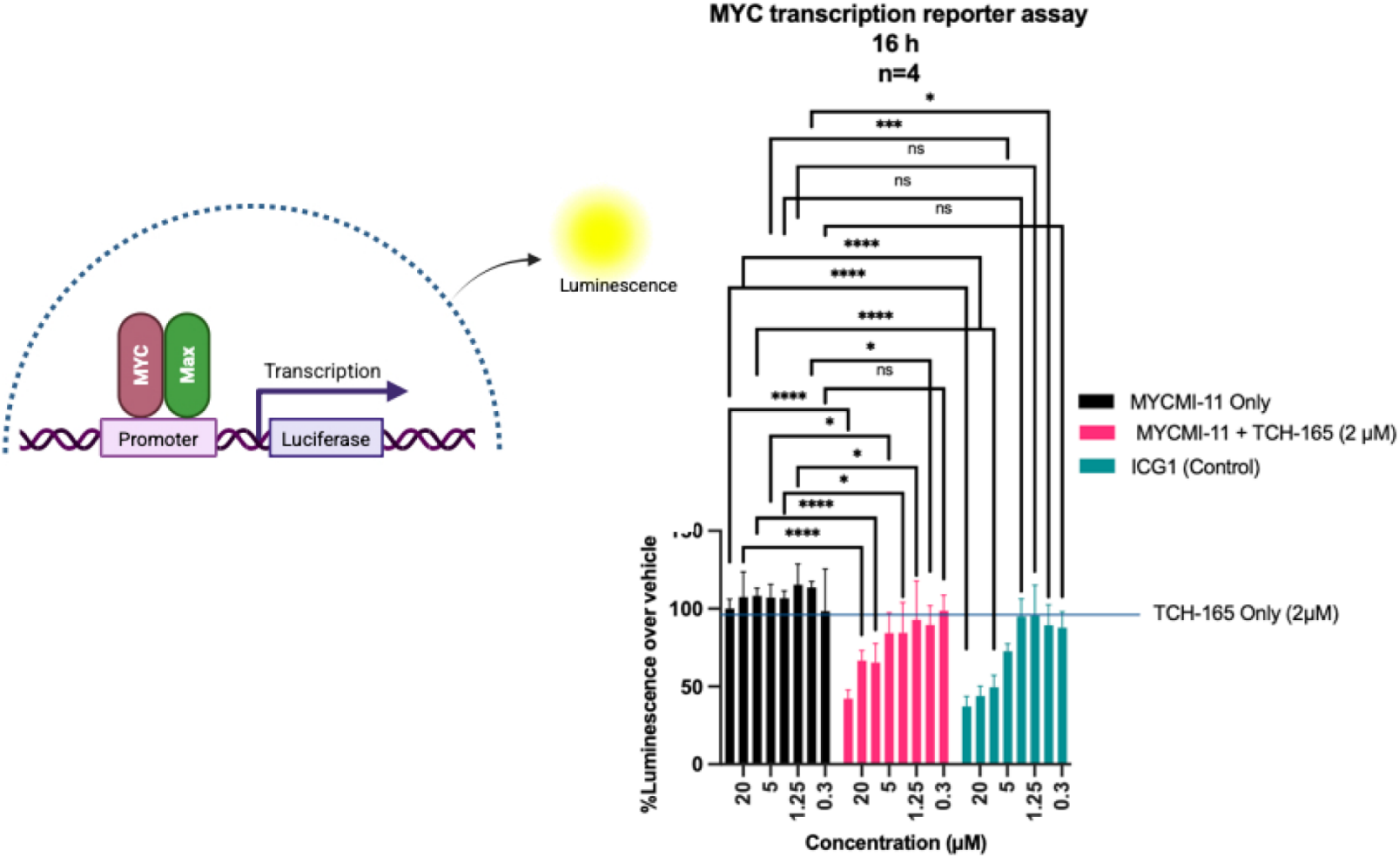
Dual targeting of MYC–MAX dimerization and 20S proteasome activation suppresses MYC transcriptional activity. HCT-116 cells stably expressing a MYC-responsive luciferase reporter were treated with increasing concentrations of MYCMI-11 (0.3–40 μM), either alone or in combination with the 20S proteasome activator TCH-165 (2 μM). Reporter activity was measured by luminescence after a 16 h period. ICG-001 (0.15–20 μM), an inhibitor of β-catenin–CBP-dependent transcription, was included as a positive control for suppression of MYC-driven transcription. Data are presented as mean ± SEM from four independent experiments (*n* = 4). Statistical significance was assessed by two-way ANOVA with Tukey’s multiple-comparisons test (*P < 0.05, **P < 0.01, ***P < 0.001, ****P < 0.0001).

Altogether, these results suggests that simultaneous disruption of MYC–MAX dimerization and activation of the 20S proteasome decreases MYC protein abundance and consequently attenuates MYC-dependent transcriptional activity. Importantly, this dual-targeting strategy highlights a potential therapeutic approach for MYC-driven cancers by simultaneously reducing MYC protein levels and disrupting MYC-dependent transcriptional programs.

### Dual Treatment with MYC–MAX Inhibitor and 20S Proteasome Activator Induces Apoptosis

To further define the cellular consequences of targeting MYC dimerization and stability, we examined whether combined MYC–MAX inhibition and 20S proteasome activation promotes apoptotic cell death in proliferating HCT-116 colon cancer cells. Given MYC’s central role in sustaining transcriptional programs that promote tumor cell survival,^2,4^ we examined whether enhanced MYC depletion induced by cotreatment translated into induction of apoptosis using Annexin V/propidium iodide (PI) staining followed by flow cytometric analysis.

Cells were treated with MYCMI-11 (10 μM), TCH-165 (10 μM), or the combination for 48 h prior to quantification of viable (Annexin V^–^/PI^–^), early apoptotic (Annexin V^+^/PI^–^), and late apoptotic (Annexin V^+^/PI^+^) populations (Figure 6). Bortezomib (300 nM), a proteasome inhibitor with established pro-apoptotic activity, served as a positive control.^43^ Whereas single-agent treatment produced only modest effects, the combination markedly increased late apoptotic fractions, accompanied by a reduction in viable cells. These findings suggest that concurrent disruption of MYC–MAX interactions and activation of the 20S proteasome impairs MYC-driven transcriptional programs that support cell survival, thereby sensitizing HCT-116 cells to apoptotic cell death. Notably, the induction of apoptosis parallels the observed reduction in MYC protein levels and transcriptional activity, supporting a functional link between MYC attenuation and loss of cellular viability. Collectively, these results are consistent with a model in which simultaneous targeting of MYC stability and dimerization shifts cellular fate toward apoptosis.

**Figure 6:**
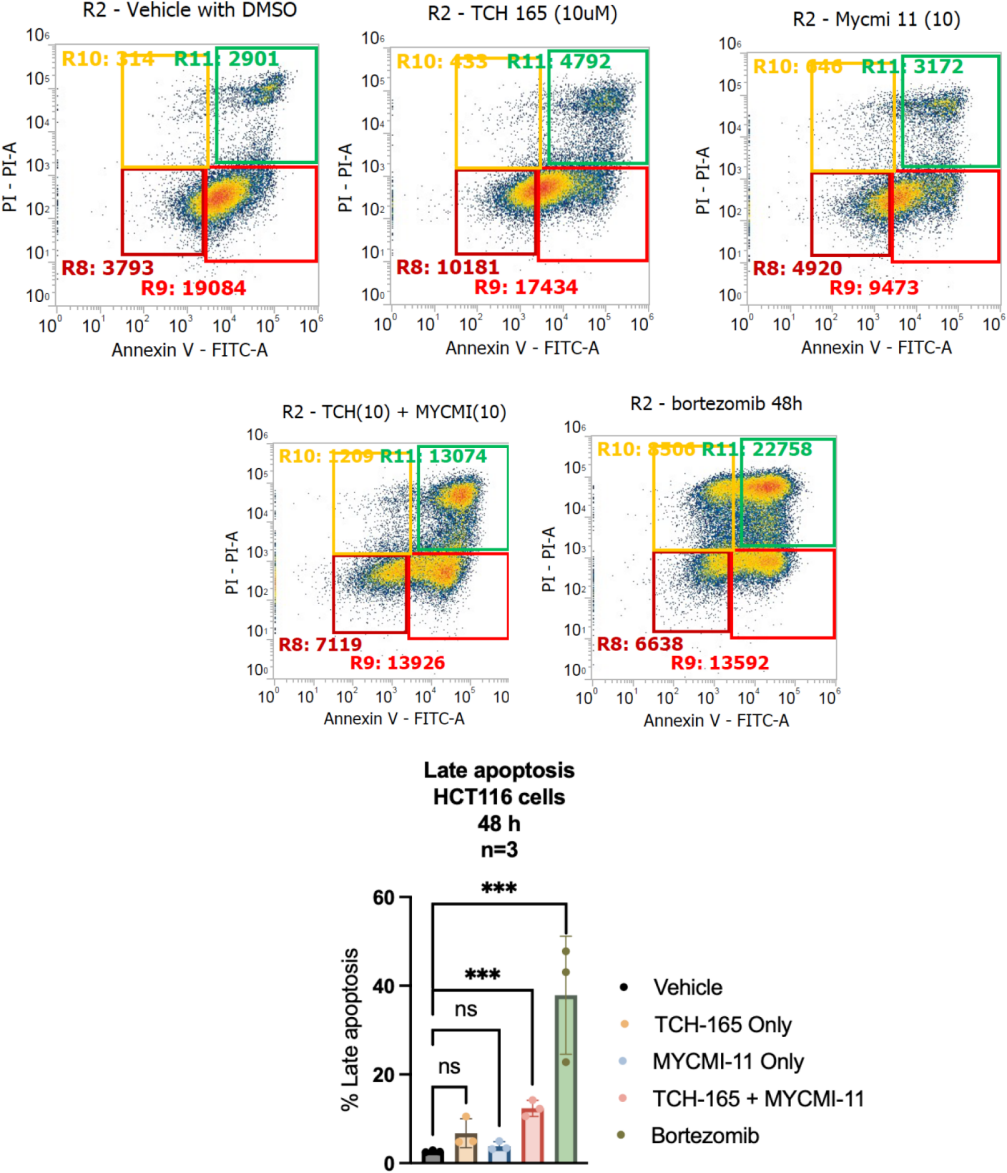
Combining MYC–MAX inhibition and 20S proteasome activation promotes apoptosis in HCT-116 cells. HCT-116 colon cancer cells were treated with DMSO (vehicle control), MYCMI-11 (10 μM), TCH-165 (10 μM), bortezomib (positive control; 300 nM), or the combination of MYCMI-11 and TCH-165 (each at 10 μM) for 48 h. Apoptosis was assessed by Annexin V/propidium iodide (PI) staining followed by flow cytometry. Representative dot plots and quantification of viable (Annexin V^–^/PI^–^), early apoptotic (Annexin V^+^/PI^–^), and late apoptotic (Annexin V^+^/PI^+^) populations (shown in the accompanying graph). The combination treatment increased apoptotic fractions relative to single agents and vehicle control. Data are presented as mean ± SD from three independent experiments (*n* = 3). Statistical significance was determined by one-way ANOVA with Dunnett’s multiple-comparisons test (*p < 0.05, **p < 0.01, ***p < 0.001).

### MYC Degradation Induced by 20S Proteasome Activation Occurs Despite Attenuation of SCF–FBXW7-Mediated Ubiquitination

The E3 ubiquitin ligase FBXW7, a component of the SCF (SKP1–CUL1–F-box) complex, is a key and well-characterized regulator of MYC ubiquitination and 26S proteasomal degradation among several E3 ligases reported to target MYC, and was selected for knockdown to evaluate its role in the context of enhanced MYC turnover.^9^ To evaluate whether the observed reduction in MYC levels requires canonical ubiquitin-dependent degradation, FBXW7 was silenced by RNA interference in HCT-116 colorectal carcinoma cells. Double transfection with FBXW7 siRNA over 48 h resulted in approximately 50% knockdown of FBXW7 and stabilization of MYC protein (Figure 7A).

**Figure 7:**
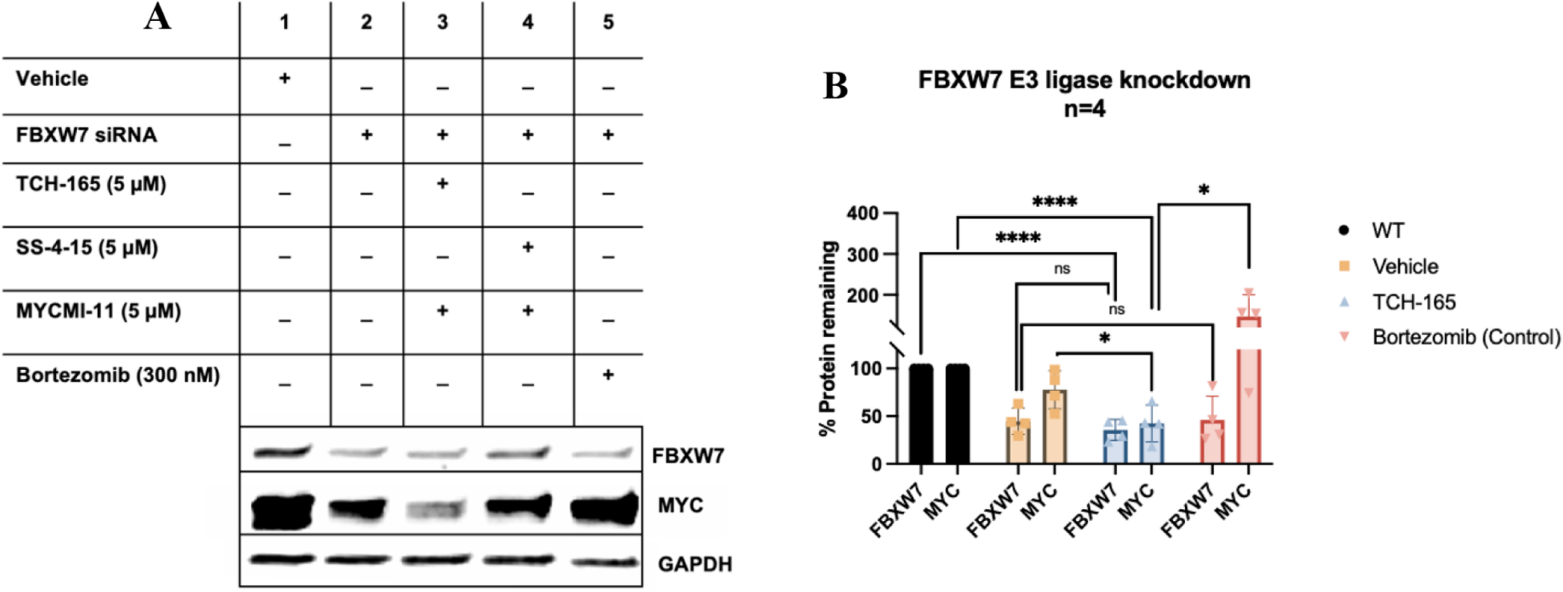
Effects of combination treatment on MYC levels following FBXW7 knock down. HCT-116 cells were subjected to siRNA-mediated silencing of FBXW7 for 48 h, followed by treatment with MYCMI-11 (5 μM) in combination with either the 20S proteasome activator TCH-165 (5 μM) or SS-4-15 (5 μM) for 4 h after transfection. (A) Immunoblot analysis of total cell lysates was performed using the indicated antibodies, with GAPDH as a loading control. (B) Quantification of remaining FBXW7 (∼70 kDa) and MYC (∼53 kDa) protein levels relative to control. Data represent mean ± SEM from three independent experiments (*n* = 4). Statistical significance was determined using two-way ANOVA followed by Šidák’s multiple comparisons test; *p* < 0.05 (), *p* < 0.01 (), *p* < 0.001 (), *p* < 0.0001 (****).

Despite partial FBXW7 depletion, treatment with the 20S proteasome activator TCH-165 (5 μM) in combination with the MYC–MAX dimerization inhibitor MYCMI-11 (5 μM) for 4 h following siRNA transfection produced a significant reduction in MYC protein levels (∼50%) (Figure 7A and B). These results indicate that MYC degradation persists despite attenuation of FBXW7-mediated ubiquitination, supporting a mechanism that is partially independent of canonical ubiquitin-dependent pathways. This is consistent with combined MYC–MAX disruption and 20S proteasome activation as a strategy to promote MYC degradation, although contributions from additional E3 ubiquitin ligases beyond FBXW7 cannot be excluded. In FBXW7 knockout cells, SS-4-15/MYCMI-11 treatment did not significantly alter MYC protein levels under these conditions.

## Discussion

Targeting the oncogenic transcription factor MYC remains challenging due to its intrinsic disorder and lack of conventional fixed pockets.^12,13^ In this study, we demonstrate that simultaneous disruption of the MYC–MAX complex and pharmacologic activation of the 20S proteasome enhances MYC degradation, suppresses MYC-dependent transcription, and promotes apoptosis in colon cancer cells. These findings support a model in which destabilization of MYC dimerization sensitizes the protein to proteasomal turnover, while 20S proteasome activation promotes its degradation.

MYC stability is tightly controlled by proteasomal pathways, most prominently through the ubiquitin-dependent degradation by the 26S proteasome.^5,6^ However, this pathway is frequently dysregulated due to alterations in the phosphorylation–ubiquitination axis that normally governs MYC turnover, leading to aberrant stabilization and accumulation of the protein. MYC also contains extensive intrinsically disordered regions that can serve as substrates for ubiquitin-independent degradation by the 20S core particle. Activation of the 20S proteasome likely enhances direct engagement of these disordered regions, facilitating MYC proteolysis independent of ubiquitin tagging. Consistent with this interpretation, proteasome inhibition attenuated MYC loss following treatment, supporting a proteasome-dependent mechanism. Thus, MYC–MAX disruption and 20S activation appear mechanistically complementary: one destabilizes functional complex formation, while the other enhances degradation of the exposed, disordered protein.

This strategy differs from E3 ligase-recruiting approaches such as PROTACs, which rely on intact ubiquitination machinery.^23^ MYC turnover is physiologically regulated in part by E3 ligases, primarily FBXW7.^9^ However, alterations in ubiquitin signaling may limit the efficiency of ubiquitin-dependent degradation strategies in certain contexts.^10,11^ By contrast, pharmacologic enhancement of 20S activity bypasses the requirement for ubiquitin conjugation,^44^ offering an alternative route to target intrinsically disordered oncoproteins.

Functionally, combined treatment produced greater suppression of MYC-driven transcription and increased apoptotic signaling compared to single agents, consistent with depletion of MYC below a critical oncogenic threshold. While formal synergy analysis was not performed, the enhanced molecular and phenotypic effects observed with cotreatment support cooperative engagement of complementary regulatory mechanisms.

Several limitations warrant consideration. These studies were conducted in a colorectal cancer model, and broader validation across other MYC-dependent systems will be necessary. Additionally, global 20S activation may influence other intrinsically disordered substrates, and proteomic profiling will be important to assess selectivity. Comprehensive CC_50_ profiling in malignant versus nonmalignant cells was also not performed and will be important in future studies to further evaluate the cytotoxicity and therapeutic window of this cooperative degradation strategy. In vivo studies will additionally be required to define the therapeutic window and durability of MYC suppression through proteasome activation. Finally, an additional limitation of this study is the use of a single MYC–MAX inhibitor, evaluation of structurally distinct MYC–MAX dimerization inhibitors, including covalent inhibitors of this interaction^45^ will be important to determine the generality of this cooperative degradation strategy, distinguish reversible versus covalent targeting mechanisms, and to exclude scaffold-specific effects.

Collectively, our findings demonstrate that orthogonal modulation of MYC–MAX dimerization and proteasomal degradation can cooperatively reduce MYC abundance and transcriptional output. This highlights pharmacological 20S activation as a complementary strategy for targeting intrinsically disordered transcription factors and expanding the chemical biology toolkit for modulating traditionally intractable oncoproteins.

## Supplementary Material



## Notes

Surface plasmon resonance (SPR) sensograms; supplementary figures; full uncropped immunoblots with corresponding densitometric quantification values; experimental materials and methods (PDF)
