# Supplementary material for: Chemical Induction of MYC Protein Degradation via MYC–MAX Disruption and 20S Proteasome Activation
Source: ACS Chem Biol. 2026 Jun 19;21(7):1728–37. doi: 10.1021/acschembio.6c00258 (PMC13386462; doi:10.1021/acschembio.6c00258)
Supplement: Supplementary file 1 [file cb6c00258_si_001.pdf]

Supplementary Material

Chemical Induction of MYC Protein Degradation via MYC–MAX Disruption and 20S Proteasome Activation

Miracle O. Olatunde,[1] *and Jetze J. Tepe*[1,2]*

**Correspondence: cew9xb@virginia.edu*

University of Virginia, Charlottesville, Virginia 22904, United States

Supplementary figures and legends

• Surface Plasmon Resonance (SPR) sensograms Page S3

• Additional figures and uncropped blots Page S5

•Materials and Methods Page S11

•^{1}H and ^{13}C NMRs of synthesized compounds Page S17

A

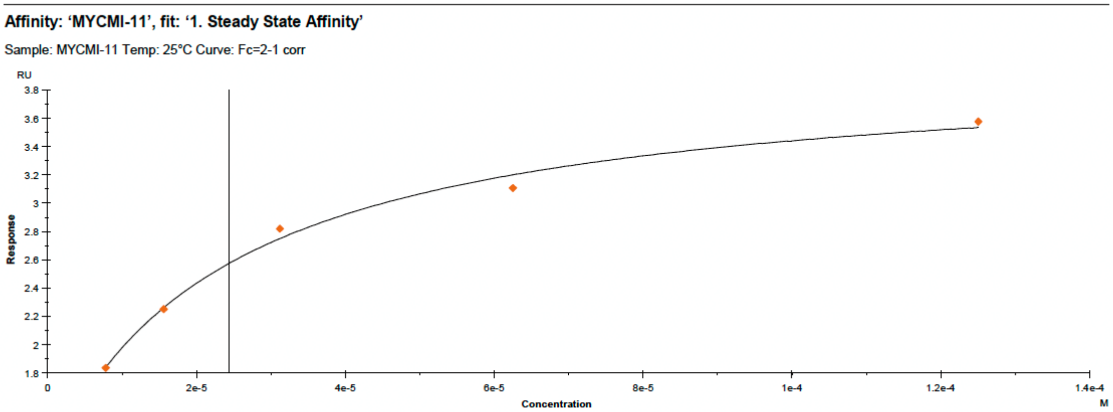
Affinity: 'MYCMI-11', fit: '1. Steady State Affinity'
Sample: MYCMI-11 Temp: 25°C Curve: Fc=2-1 corr
RU
Response
Concentration
M
3.8
3.6
3.4
3.2
3
2.8
2.6
2.4
2.2
2
1.8
0
2e-5
4e-5
6e-5
8e-5
1e-4
1.2e-4
1.4e-4

B

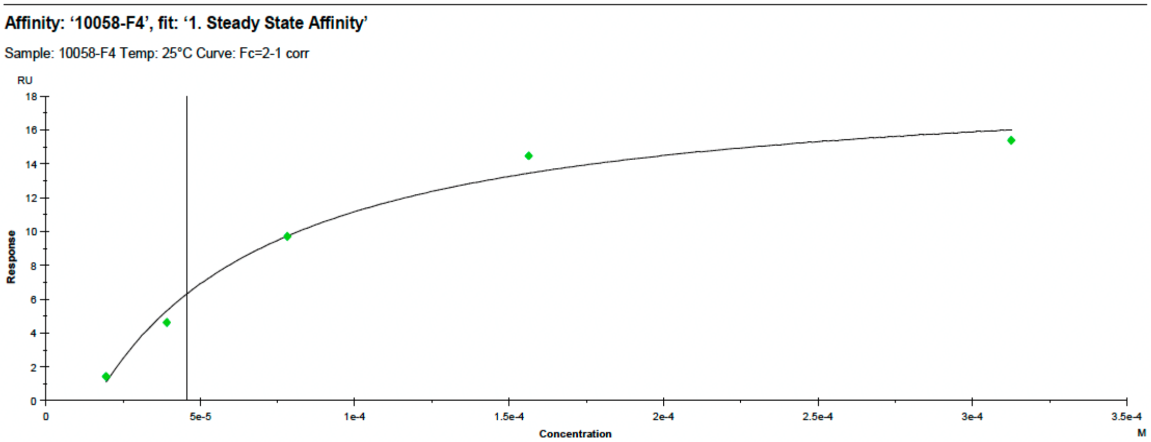
Affinity: '10058-F4', fit: '1. Steady State Affinity'
Sample: 10058-F4 Temp: 25°C Curve: Fc=2-1 corr
RU
Response
Concentration
M
18
16
14
12
10
8
6
4
2
0
0
5e-5
1e-4
1.5e-4
2e-4
2.5e-4
3e-4
3.5e-4

C

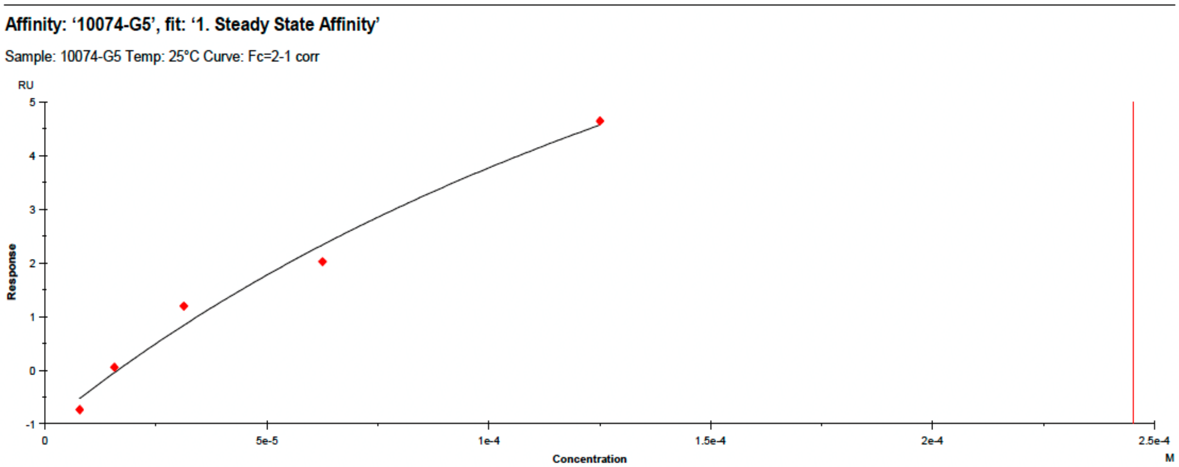
Affinity: '10074-G5', fit: '1. Steady State Affinity'
Sample: 10074-G5 Temp: 25°C Curve: Fc=2-1 corr
RU
Response
Concentration
M
5
4
3
2
1
0
-1
0
5e-5
1e-4
1.5e-4
2e-4
2.5e-4

D

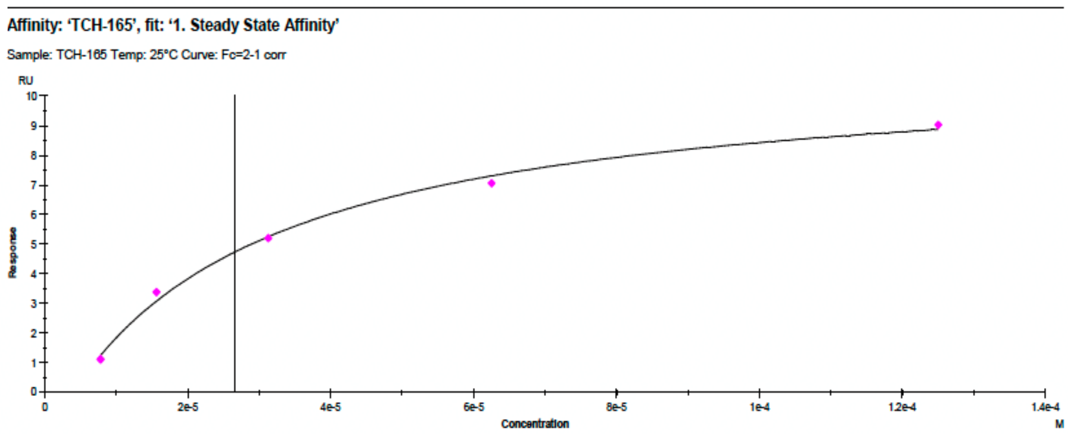

E

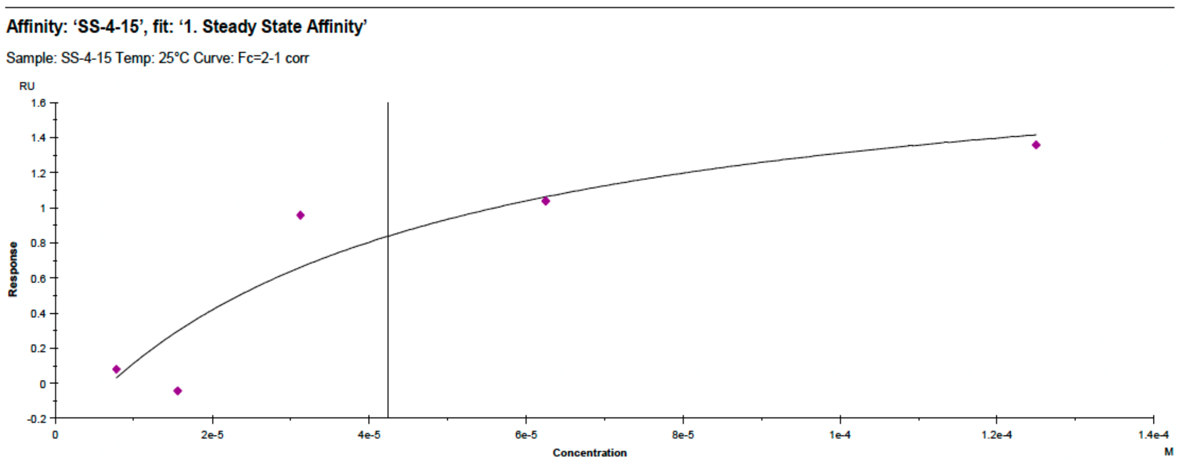

Figure S1. Equilibrium binding of small molecules to the c-MYC bHLHZip domain.

Surface Plasmon Resonance analysis of MYC-Max inhibitors: MYCMI-11 (A), 10058-F4 (B), 10074-G5 (C), and proteasome activators: TCH-165 (D), and SS-4-15 (E) at the indicated concentrations at 25°C. All compounds were prepared in running buffer containing 5% DMSO.

Response units are shown after solvent correction and reference subtraction. Data are representative of three independent experiments with different immobilizations. Sensograms were reference subtracted and globally fitted to a 1:1 Langmuir binding model, consistent with a simple bimolecular interaction between immobilized MYC fragment and the small molecule analytes.

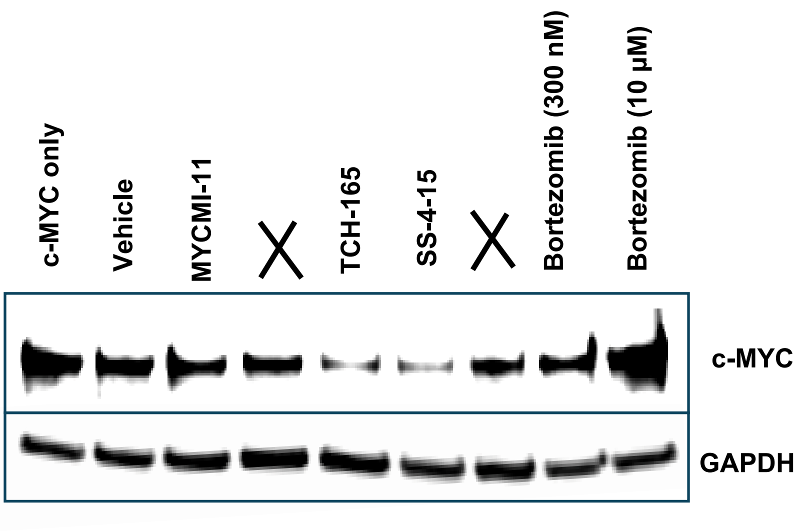

Figure S2. In-vitro MYC degradation through 20S proteasome activation. (A) Purified human 20S proteasome (10 nM) was incubated with purified monomeric MYC (79 nM) at 37 °C for 4 h in the presence of 10 µM of proteasome activators including SS-4-15, TCH-165, or MYC-MAX inhibitor, MYCMI-11. Bortezomib (10 µM) was included as a proteasome inhibitor control. MYC levels were analyzed by immunoblotting, with purified GAPDH used as a loading control, and densitometric quantification is shown in accompanying graph. Lanes 4 and 7 were included from preliminary screening and are not relevant to the conclusions of this manuscript.

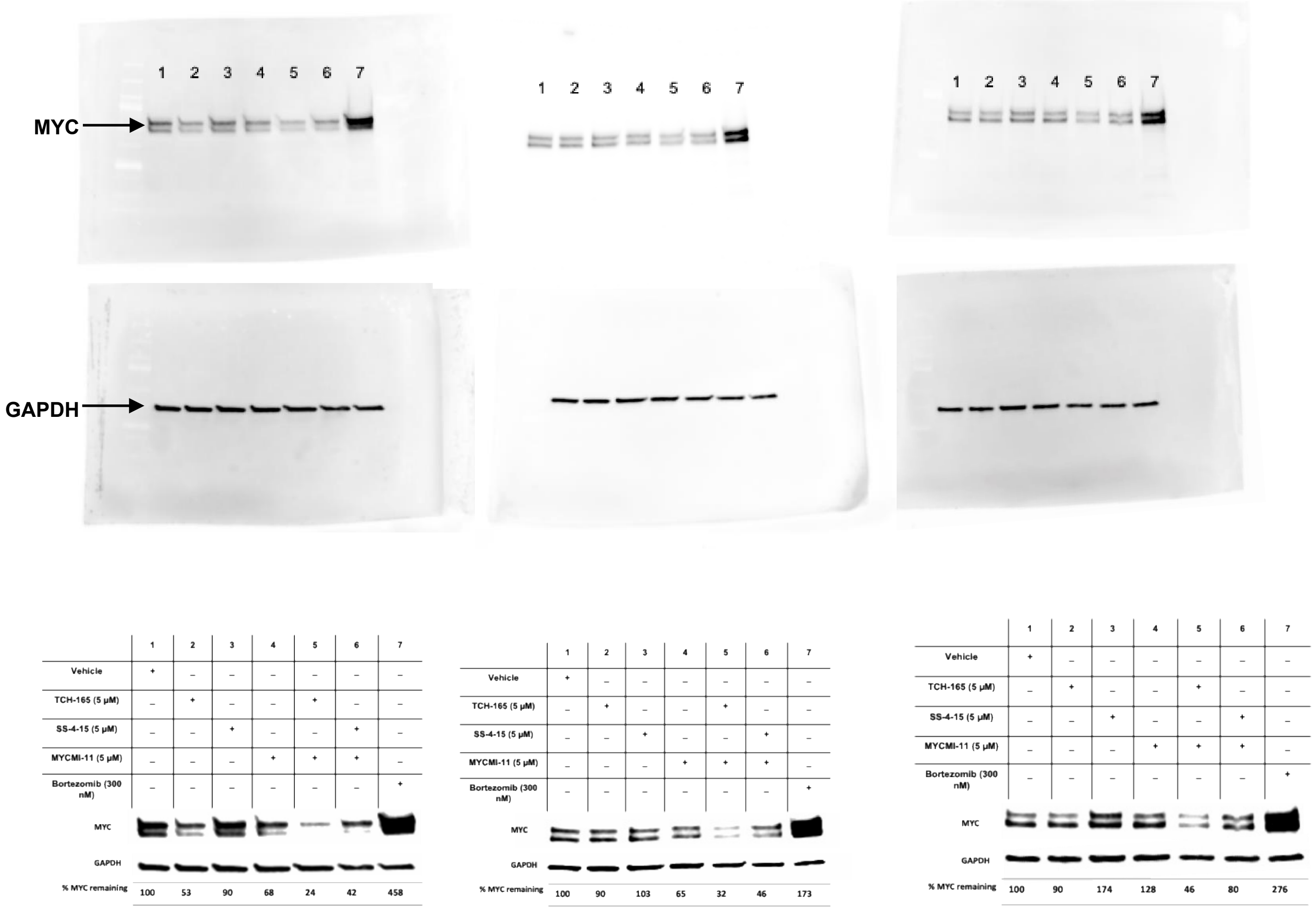

Figure S3. Full immunoblots for MYC degradation in HT-29 cells supporting figure 4B. HT-29 cells were treated for 4 h with MYCMI-11 (5 µM), TCH-165 (5 µM), SS-4-15 (5 µM), or combinations as indicated. Whole-cell lysates were analyzed by immunoblotting for MYC (~53 kDa), with GAPDH (~36 kDa) as a loading control. Full, uncropped blots are shown. Corresponding analyzed, cropped blot with their analyzed density numbers are also shown.

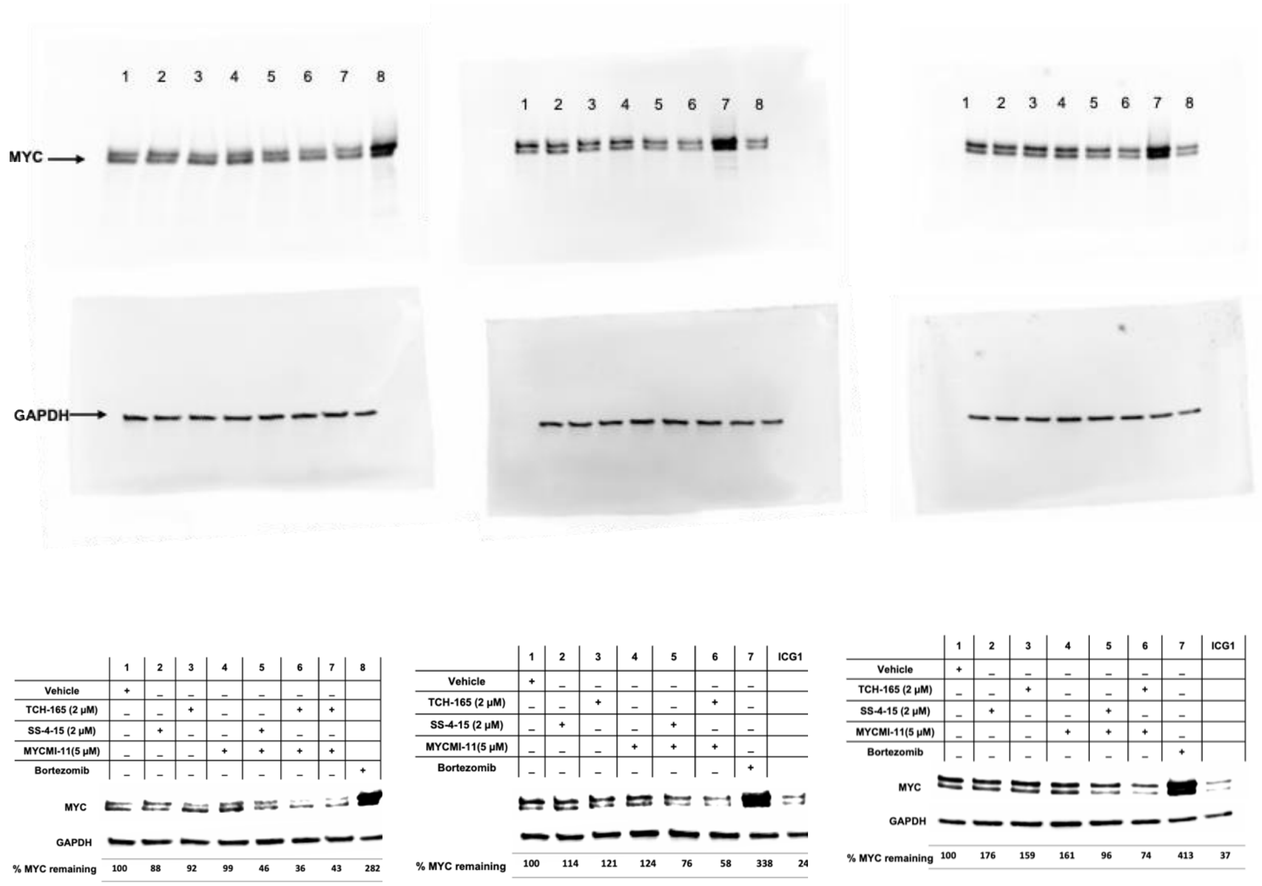

Figure S4. Full immunoblots for MYC degradation in HCT-116 cells supporting figure 4A. HCT-116 cells were treated for 4 h with MYCMI-11 (5 µM), TCH-165 (2 µM), SS-4-15 (2 µM), or combinations as indicated. Whole-cell lysates were analyzed by immunoblotting for MYC (~53 kDa), with GAPDH (~36 kDa) as a loading control. Full, uncropped blots are shown. Corresponding analyzed, cropped blot with their analyzed density numbers are also shown.

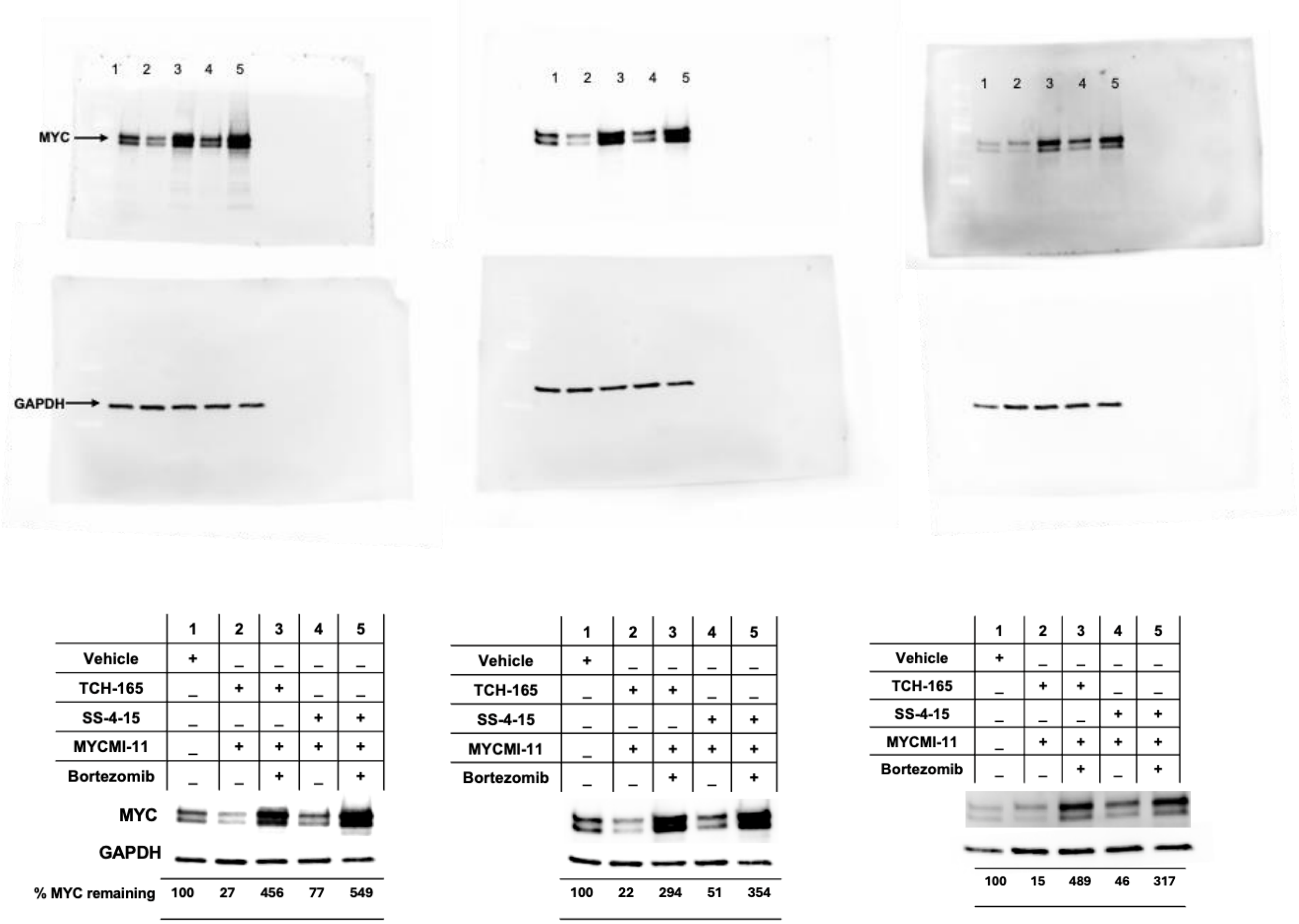

Figure S5. Proteasome inhibition experiments. Uncropped immunoblots supporting Figure 4C. HT-29 cells were pre-treated with bortezomib (300 nM) for 1 h prior to combination treatment. then with the combination of MYCMI-11 (5 µM), and TCH-165 (5 µM), or SS-4-15 (5 µM), as indicated. Whole-cell lysates were probed for MYC (~53 kDa) with GAPDH (~36 kDa) as a loading control. Full-length, uncropped immunoblots are provided. Corresponding analyzed cropped blot with their analyzed density numbers are also shown.

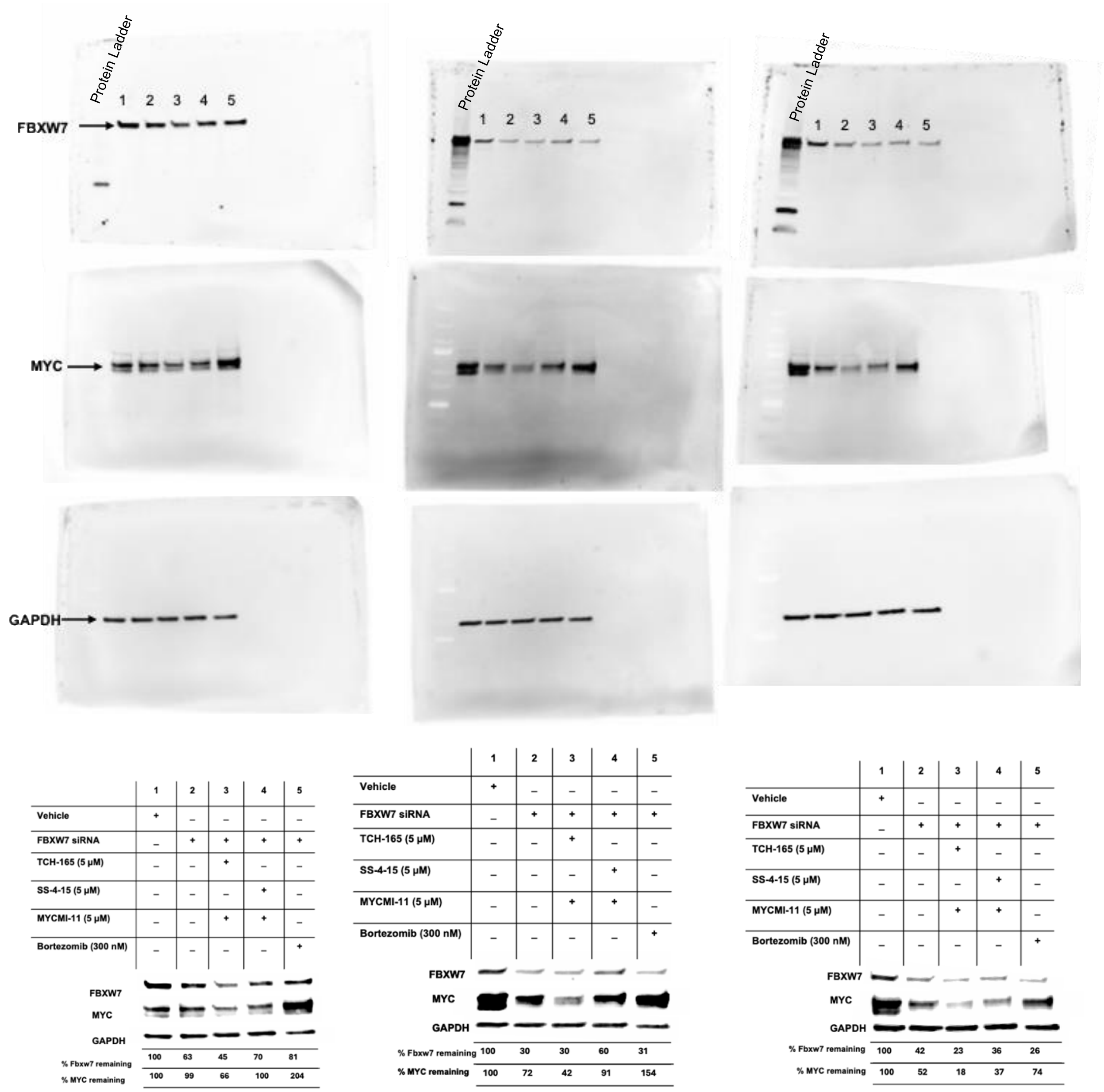

Figure S6. FBXW7 siRNA knockdown experiment. Full raw blots supporting figure 7. HCT-116 cells were subjected to siRNA-mediated silencing of FBXW7 for 48h, followed by treatment with MYCMI-11 (5 µM) in combination with the 20S proteasome activator TCH-165 (5 µM) or SS-4-15 (5 µM) for 4h after transfection. Immunoblot analysis of total cell lysates was performed using the indicated antibodies, with GAPDH (~36 kDa) as a loading control. Corresponding analyzed cropped blot with their analyzed density numbers are also shown of

remaining FBXW7 (~70kDa) and MYC (~53 kDa) protein levels relative to loading control are also shown.

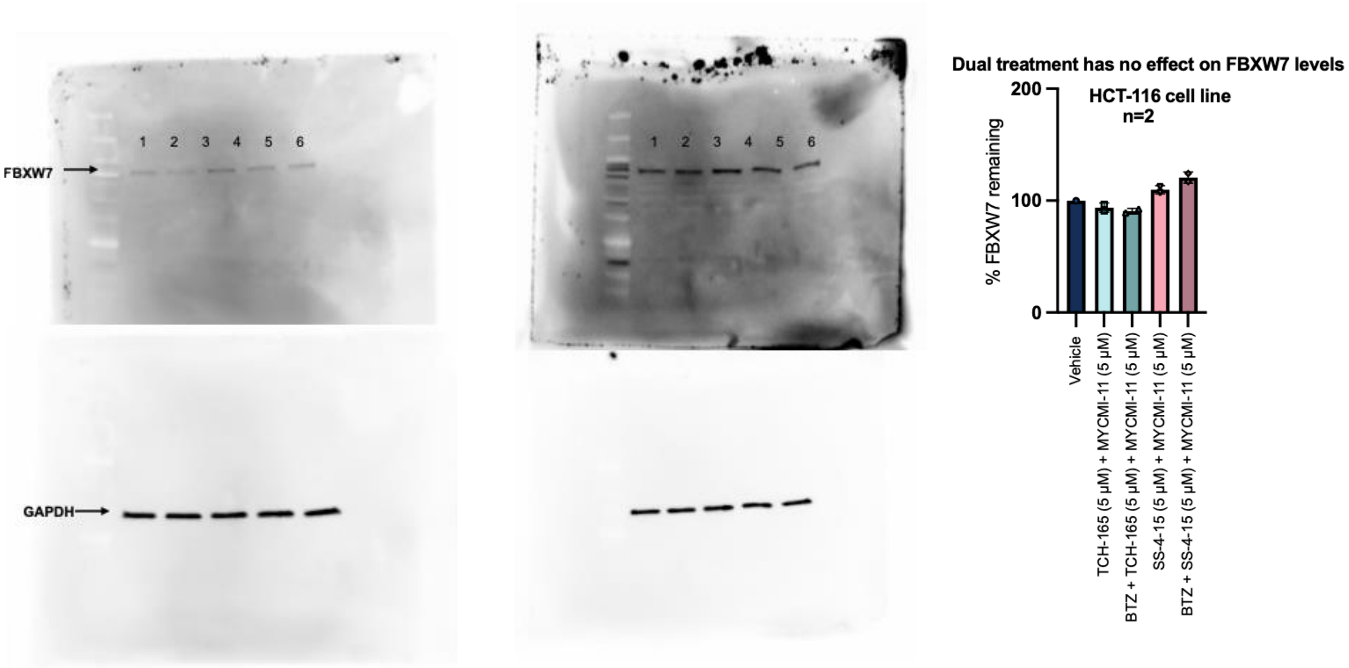

Figure S7. Dual treatment has no effect on FBXW7 levels without siRNA treatment. Uncropped blots supporting Figure 7. HCT-116 cells were subjected to treatment with MYCMI-11 (5 µM) in combination with the 20S proteasome activator TCH-165 (5 µM) or SS-4-15 (5 µM) for 4h (or with bortezomib) as indicated. Immunoblot analysis of total cell lysates was performed using the indicated antibodies, with GAPDH (~36 kDa) as a loading control. Corresponding graph of analyzed density numbers of remaining FBXW7 (~70kDa) protein levels relative to loading control are also shown.

Materials and Methods

Human 20S proteasome (R&D Systems/Bio-Techne, catalog #E-360) was used, along with the fluorogenic peptide substrates N-succinyl-Leu-Leu-ValTyr-7-amido-4-methylcoumarin, Suc-LLVY-AMC (Cayman Chemicals, catalog #10008119) carboxyl benzyl-Leu-Leu-Glu-7-amido-4-methylcoumarin, Z-LLE-AMC (Cayman Chemicals, catalog #10008117) and tert-butyloxycarbonyl-Leu-Arg-Arg-7-amido-4-methylcoumarin, Boc-LRR-AMC (Cayman Chemicals, catalog #26642). For SPR experiments, CM5 sensor chips (Cytiva, catalog #BR100012) and GST Capture Kit (Cytiva, catalog, #BR100223), amine coupling kit (Cytiva, catalog, #BR100050),were employed. Cell viability used Cell Titer-Glo ® (Promega, catalog #G8461) and ONE-Step™ Luciferase Assay System Reagent (BPS Bioscience, catalog #60690). Antibodies: rabbit anti-cMyc (Abcam, catalog #ab32072), anti-FBXW7 (Abcam, catalog #ab192328).

In Vitro Fluorogenic Peptide Degradation - 20S Proteasome Activation Assay

A total of 89 µL of 1.12 nM 20S proteasome in 38 mM Tris–HCl containing 100 mM NaCl (pH 7.8) was dispensed into each well of a black, clear-bottom 96-well plate. Subsequently, 1 µL of compound from a serial dilution plate was added to yield a starting concentration of 80 µM. For blank wells, 89 µL of assay buffer and 1 µL of DMSO were added. The plate was incubated for 15 min at 37 °C. Following incubation, 10 µL of a fluorogenic peptide substrate mixture: Suc-LLVY-AMC, Z-LLE-AMC, and Boc-LRR-AMC was added to achieve a final total substrate concentration of 20 µM, enabling measurement of CT-L, Casp-L, and T-L activities, respectively. Fluorescence from released AMC was recorded kinetically every 5 min for 1 h using a SpectraMax

M5e plate reader (excitation 380 nm, emission 460 nm). Initial rates were determined from the linear portion of the progress curve. The final DMSO concentration in all wells was 1%.

In vitro recombinant human MYC degradation assay

The total reaction volume was 25 μL, consisting of 22 μL of purified 20S proteasome (final concentration 10 nM) in HEPES buffer with DTT, pH 7.4, and the test compound at the indicated concentrations. Reactions were incubated at 37°C for 45 minutes. Subsequently, 2.5 μL of purified cMyc (final concentration 79 nM) was added, and the mixture was further incubated in a water bath at 37°C for 4 hours.

After incubation, 5 μL of concentrated SDS loading buffer was added, and samples were boiled for 20 minutes. Purified GAPDH (0.5 μL, 250 nM) was then added, and the samples were boiled for an additional 20 minutes. Proteins were resolved on 4–20% SDS-PAGE gels and immunoblotted with anti-cMyc antibody (1:1000), HRP-conjugated anti-rabbit IgG (1:1000), and GAPDH (6C5cc) Mouse mAb (HRP Conjugate). Blots were developed using Radiance Western reagent (Azure Biosystems, #AC2204) and imaged with an Azure imager.

Cell Culture

HCT-116, stably transfected HCT-116 MYC reporter cells, and HT-29 colon cancer cells were cultured in McCoy's 5A medium (Gibco #16600082) supplemented with 10% fetal bovine serum (FBS) and 1% penicillin-streptomycin (Pen/Strep). All cells were incubated at 37 °C in a humidified atmosphere containing 5% CO_2 and routinely tested for mycoplasma contamination.

Cell Lysis and Western Blotting

Cells were pelleted and washed with cold PBS (2X). After which they were resuspended in lysis buffer (8 mL of cold PBS + 1 tablet protease inhibitor cocktail (Roche #11836170001) and sonicated and allowed to sit on ice for 10 minutes. Cells were then spined down at 1500 g for 30 minutes at 4 °C. Total protein in the lysate was quantified using the bicinchoninic acid assay (BCA) and normalized to 2 mg/mL. Lysate was resolved on a 4-20% Tris/Glycine gel (PAGE) and transferred to a nitrocellulose membrane. The membrane was probed with anti-cMyc and anti-GAPDH antibodies. Blots were imaged using Azure imager after being developed with Radiance Western reagent.

Cellular MYC Degradation

HCT-116 and HT-29 cells were grown to about 80% confluency in McCoy's 5A (modified) media supplemented with 10 % FBS and 1% penicillin/streptomycin (250,000 cells/mL in a 6cm plate. Cells were treated with DMSO, Bortezomib or TCH-165/SS-4-15 and/or MYCMI-11 and allowed to incubate for 4 hours at 37°C under 5% CO2 . Cells were then pelleted and washed with cold PBS (2X). After which they were resuspended in lysis buffer (8 mL of cold PBS + 1 tablet protease inhibitor cocktail) and sonicated and allowed to sit on ice for 10 minutes. Cells were then spined down at 1500 g for 30 minutes at 4 °C. Total protein in the lysate was quantified using the bicinchoninic acid assay (BCA) and normalized to 1 mg/mL. Lysate was resolved on a 4-20% Tris/Glycine gel (PAGE) and transferred to a nitrocellulose membrane. The membrane was probed with anti-cMyc and anti-GAPDH antibodies. Blots were imaged using Azure imager after being developed with Radiance Western reagent.

Myc-Luciferase Reporter Assay

HCT-116 MYC reporter cells were seeded at 25,000 cells/well in a white, clear, flat- bottom 96 well plate using McCoy's 5A media supplemented with 10% FBS and 1% penicillin/streptomycin. The cells were incubated overnight at 37°C under 5% CO2. The next day, media was taken out and replaced with Opti-MEM I supplemented with 0.5% FBS, 1mM sodium pyruvate, and 1% non-essential amino acid and containing compounds (2 μM proteasome activator and (40, 20, 10, 5, 2.5, 1.3, 0.6, or 0 μM) MYCMI-11 or DMSO) . The cells were incubated for 16 hours at 37°C under 5% CO2 . One step luciferase reagent was used to measured firefly luminescence.

Apoptosis assay

Apoptosis was quantified by flow cytometry using Annexin V–FITC and propidium iodide (PI). 1×10^6 cells in a 6 cm plate were treated with the indicated compounds, and untreated cells served as negative controls. Following treatment, cells were harvested, washed once with cold phosphate-buffered saline (PBS), and resuspended in 1× annexin-binding buffer. Cell suspensions were adjusted to 1×10^6 cells/mL, and 100 μL aliquots were transferred to flow cytometry tubes. Annexin V–FITC (5 μL) and PI (1 μL of a 100 μg/mL working solution) (Thermofisher Scientific catalog #V13242) were added to each sample, followed by incubation for 15 min at room temperature in the dark. After incubation, 400 μL of 1× annexin-binding buffer was added, and samples were kept on ice until analysis. Samples by flow cytometry and compensation was performed using single-stained and unstained controls to correct for spectral overlap between FITC and PI.

FBXW7 siRNA Transfection

HCT-116 cells were seeded in 6-well plates at a density of 200,000 cells per well (100,000 cells/mL) in 2 mL of antibiotic-free culture medium and allowed to reach approximately 60% confluency. For siRNA transfection, ON-TARGETplus Human FBXW7 siRNA (Horizon, catalog #LQ-004264-00-0005) was diluted in 190 µL of serum-free Opti-MEM and incubated for 5 min at room temperature. In parallel, 10 µL of Lipofectamine™ RNAiMAX (ThermoFisher Scientific, catalog #13778100) was diluted in 190 µL of Opti-MEM and incubated for 5 min. The two solutions were combined, gently mixed, and incubated for 20 min at room temperature to allow complex formation. After 16 hours of cell attachment, the culture medium was replaced with 1.6 mL of antibiotic-free medium per well, and the siRNA–lipid complexes (390 µL total) were added, bringing the final volume to 2 mL and yielding a final siRNA concentration of 25 nM. Cells were incubated for 24 hours, after which the transfection procedure was repeated. Cells were incubated for an additional 48 hours post-transfection, followed by 4 hours of compound treatment, after which cells were lysed and protein was extracted and then quantified with BCA for Western blot analysis.

Surface Plasmon Resonance assay

Biacore X100 instrument (Cytiva). GST-tagged recombinant human c-MYC (N-terminal) protein (Novus Bio-Techne, catalog #H00004609-Q01-25 µg) was immobilized on CM5 sensor chips via capture with a polyclonal goat anti-GST antibody. The sensor surface was first activated with 100 mM N-hydroxysuccinimide (NHS) and 400 nM 1-ethyl-3-(3-dimethylaminopropyl)carbodiimide (EDC) following the manufacturer's protocol. Anti-GST was immobilized on both flow channels, F1 (reference) and F2, to approximately 5000 RU, and

remaining reactive groups were blocked with 1 M ethanolamine-NaOH (pH 8.5). GST-MYC was then captured on flow channel 2 (F2; ~1200 RU), while flow channel 1 (F1) received GST alone as the reference surface.

Small molecules were injected at varying concentrations prepared in running buffer (20 mM $Na_2HPO_4 \cdot 2H_2O$, 4 mM KH_2PO_4, 300 mM NaCl, 0.05% Surfactant P20, and 5% DMSO, pH 7.5). DMSO standard curves were generated and applied for solvent correction to compensate for DMSO-dependent bulk refractive index Association rate constants (k_a), dissociation rate constants (kd), and equilibrium dissociation constants (KD) were obtained by globally fitting the reference-subtracted sensograms (F2 – F1) to a 1:1 Langmuir binding model using BIAevaluation software. All data shown represent reference-subtracted sensograms to account for nonspecific binding.

Synthesis

4-chloro-1-methyl-1*H*-pyrazolo[3,4-*d*]pyrimidine (1)

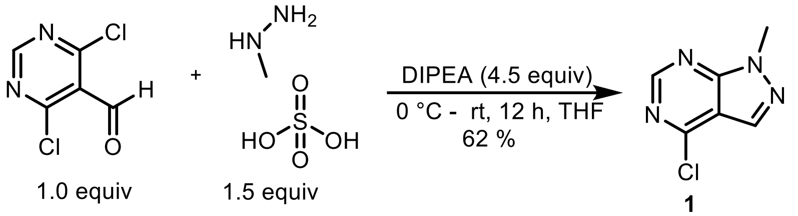

To a 100 mL round bottom flask (rbf) was added the dichloropyrimidine aldehyde (1.10g , 6.00 mmol) in tetrahydrofuran (THF), followed by the addition of diisopropylethylamine (DIPEA)(4.7 mL, 27.00 mmol) at 0 °C. The mixture was allowed to stir for 10 minutes after which methyl hydrazine sulphate (1.3 g, 9.0 mmol) was added, stirred, and allowed to cool to room temperature for 5 h after which the reaction was quenched with saturated ammonium chloride solution. The organics were isolated with dichloromethane (DCM) and washed with water (30 mL X 3). The organic layer was dried over sodium sulphate and the solvent was evaporated under reduced pressure. The product, a white solid, was isolated and purified with Combi Flash chromatography (silica gel, 20−40 µm, gradient 0−20% ethyl acetate – hexane) in 62% yield (626 mg). **MP:** 149-164°C. **^{1}H NMR** (400 MHz, CD_2Cl_2) δ 8.78 (s, 1H), 8.17 (s, 1H), 4.15 (s, 3H).**^{13}C{^{1}H} NMR** (101 MHz, CD_2Cl_2) δ 154.5, 154.4, 153.3, 131.6, 113.5, 34.3. **HRMS (APCI) m/z:** Calculated for $C_6H_6ClN_4^+$ $[M+H]^+$ 169.0276 found 169.0277. **IR (cm^{-1}):** 3345, 1951, 1908, 1395.

***N*-butyl-1-methyl-*N*-phenyl-1*H*-pyrazolo[3,4-*d*]pyrimidin-4-amine (2)**

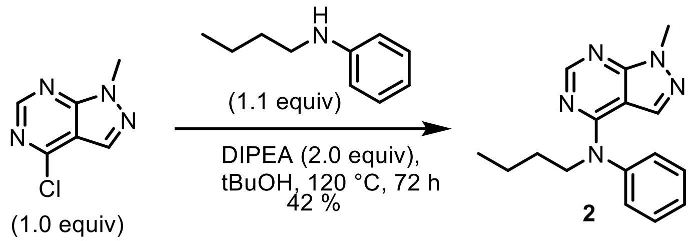

To a solution of compound **1** (169.0 mg, 1.0 mmol) in t-butanol (2mL) was added N-butylaniline (0.18 mL, 1.1 mmol), followed by DIPEA (0.35 mL, 2.0 mmol). The reaction was allowed to stir at 120 °C for 72 h in a sealed tube (reflux). The product, a white solid, was isolated and purified with Combi Flash chromatography (silica gel, 20−40 µm, gradient 0−40% ethyl acetate – hexane) in 42% yield (119 mg). **MP:** 83 – 89 °C. **^{1}H NMR** (400 MHz, CD_2Cl_2) δ 8.41 (s, 1H), 7.64 – 7.48 (m, 3H), 7.41 – 7.30 (m, 2H), 5.90 (s, 1H), 4.16 – 4.04 (m, 2H), 3.88 (s, 3H), 1.80 – 1.63 (m, 2H), 1.41 (dq, *J* = 14.8, 7.4 Hz, 2H), 0.96 (t, *J* = 7.4 Hz, 3H). **^{13}C{^{1}H} NMR** (101 MHz, CD_2Cl_2) δ 157.6, 155.5, 154.1, 143.9, 132.7, 130.6, 129.5, 129.0, 101.4, 50.7, 33.9, 30.0, 20.5, 14.1. **HRMS (APCI) m/z:** Calculated for $C_{16}H_{20}N_5^+$ $[M+H]^+$ 282.1713 found 282.1714. **IR (cm^{-1}):** 3009**,** 2930, 1558.5, 1381.f

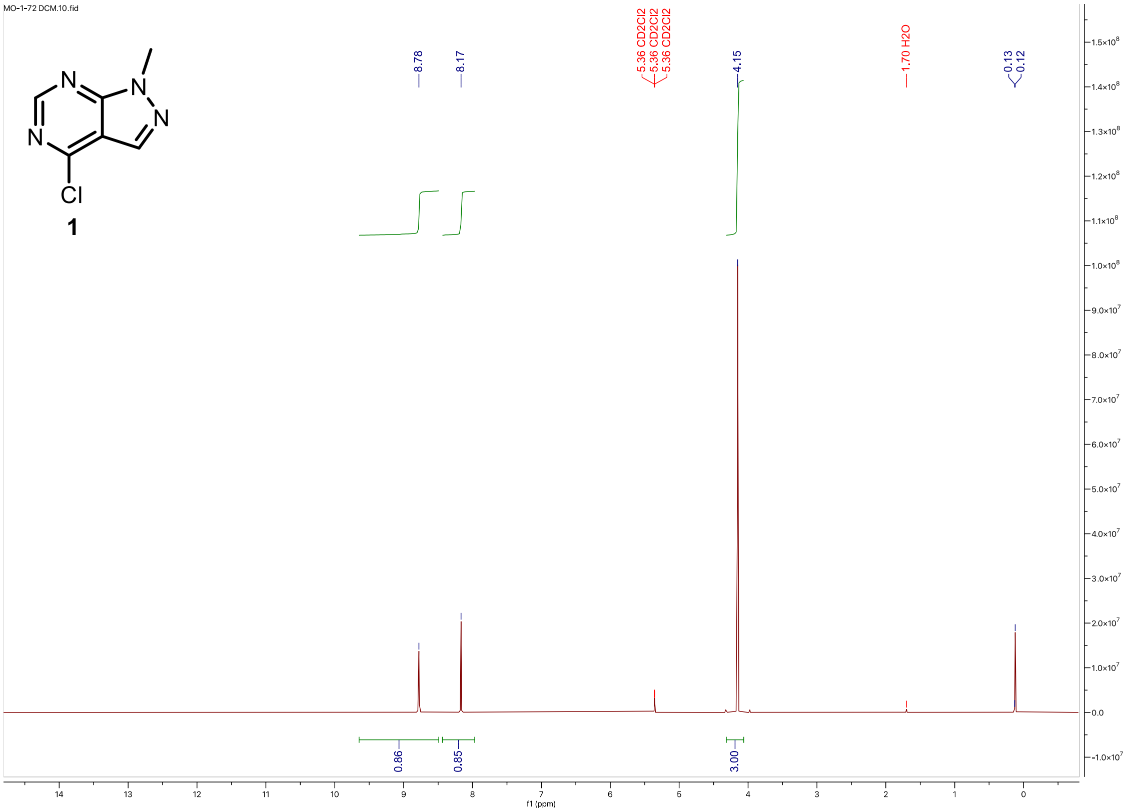
MO-1-72 DCM.10.fid
1
8.78
8.17
5.36 CD2Cl2
5.36 CD2Cl2
5.36 CD2Cl2
4.15
1.70 H2O
0.13
0.12
0.86
0.85
3.00
f1 (ppm)

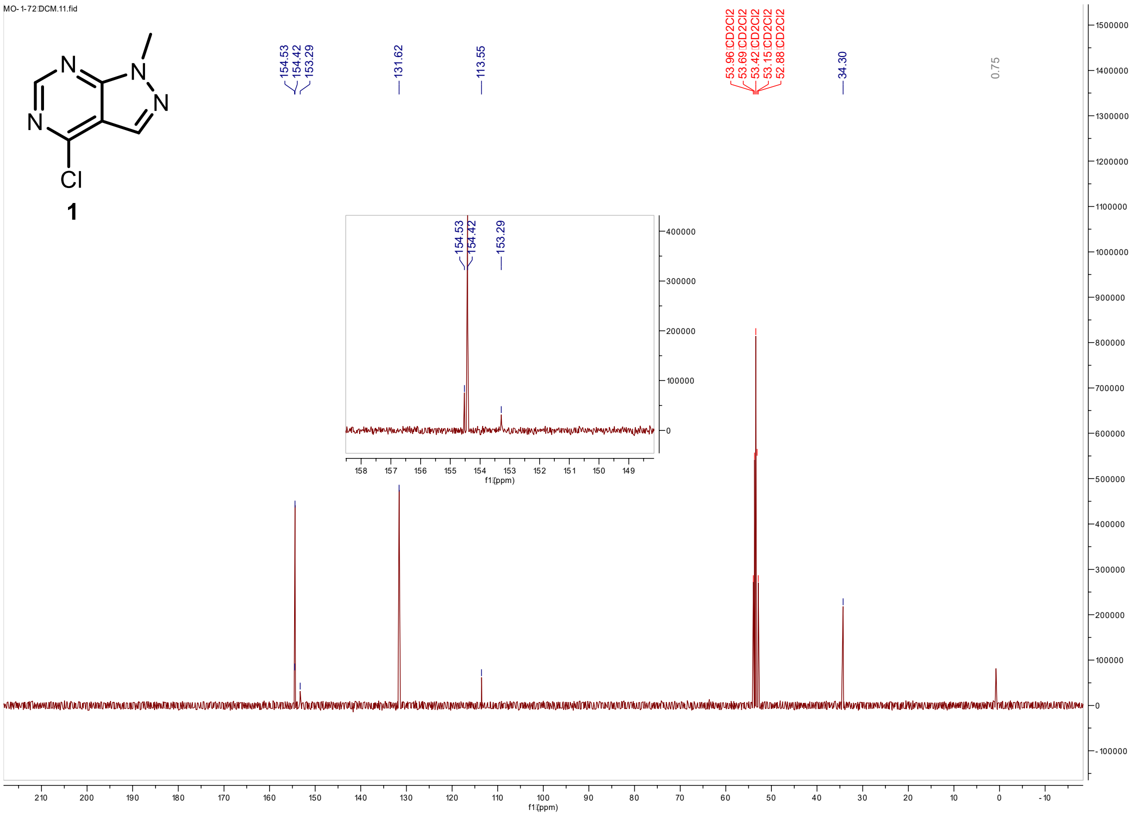
MO- 1-72 DCM.11.fid
1
154.53
154.42
153.29
131.62
113.55
53.96 CD2Cl2
53.69 CD2Cl2
53.42 CD2Cl2
53.15 CD2Cl2
52.88 CD2Cl2
34.30
0.75
f1 (ppm)

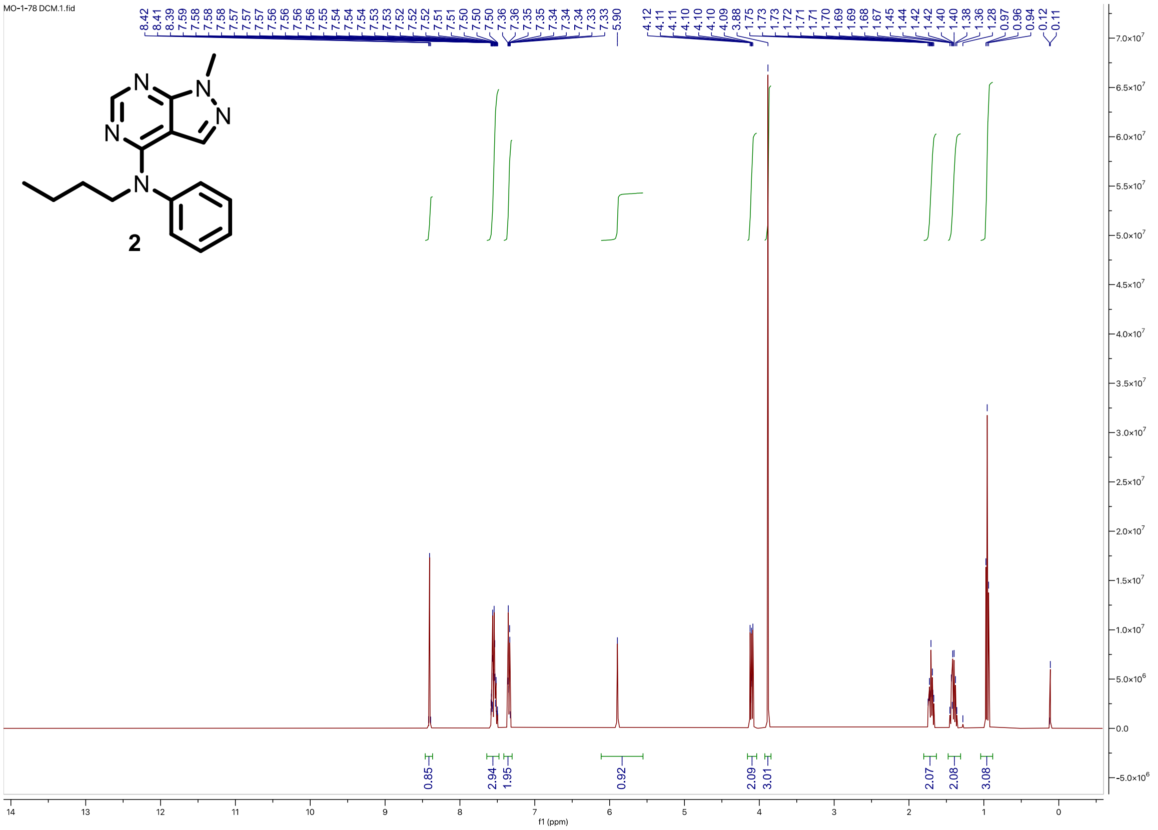
MO-1-78 DCM.1.fid
8.42 8.41 8.39 7.59 7.58 7.58 7.57 7.57 7.57 7.56 7.56 7.56 7.55 7.54 7.54 7.54 7.53 7.53 7.52 7.52 7.52 7.51 7.51 7.50 7.50 7.50 7.36 7.36 7.35 7.35 7.34 7.34 7.34 7.33 7.33 5.90 4.12 4.11 4.10 4.10 4.10 4.09 3.88 1.75 1.73 1.73 1.72 1.71 1.71 1.70 1.69 1.69 1.68 1.67 1.45 1.44 1.42 1.42 1.40 1.40 1.38 1.36 1.28 0.97 0.96 0.94 0.12 0.11
2
0.85 2.94 1.95 0.92 2.09 3.01 2.07 2.08 3.08
f1 (ppm)

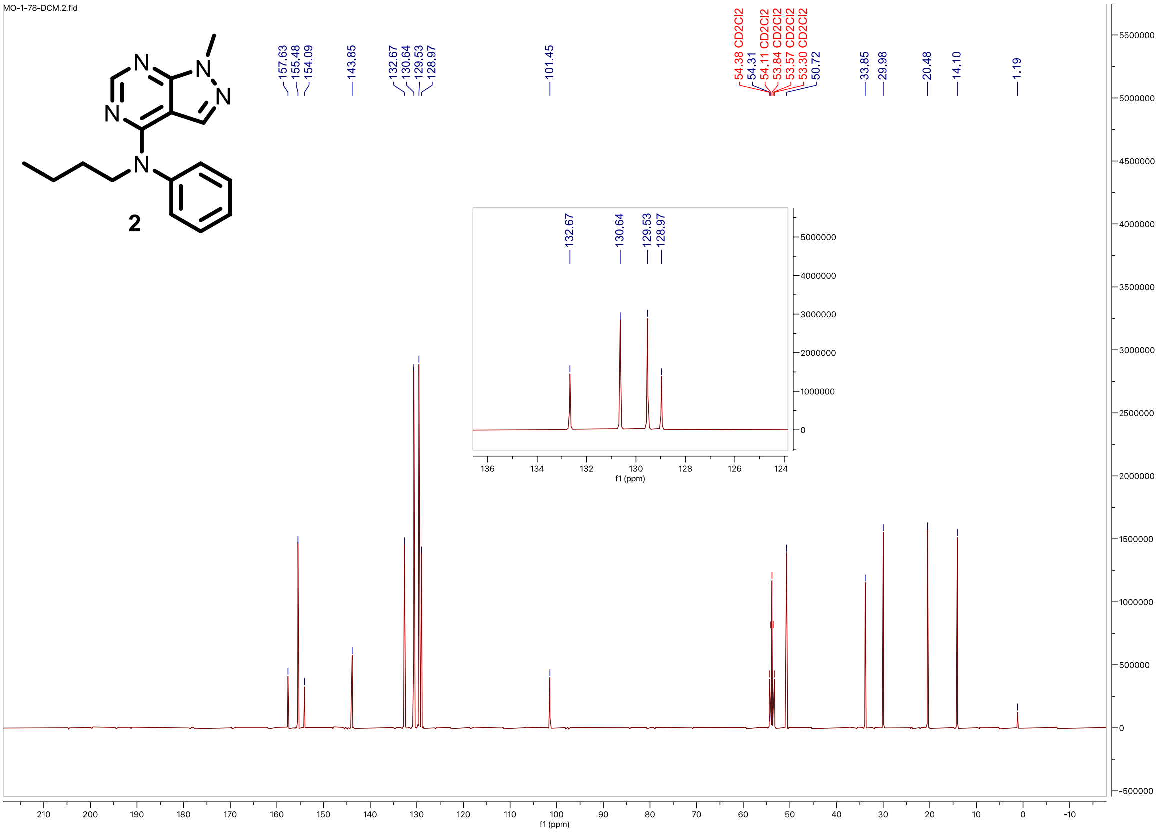
MO-1-78-DCM.2.fid
157.63 155.48 154.09 143.85 132.67 130.64 129.53 128.97 101.45
54.38 CD2Cl2 54.31 54.11 CD2Cl2 53.84 CD2Cl2 53.57 CD2Cl2 53.30 CD2Cl2 50.72
33.85 29.98 20.48 14.10 1.19
2
132.67 130.64 129.53 128.97
f1 (ppm)
